# Regionally extensive ejecta layer of the Australasian tektite strewn field: the MIS 20/19 large meteorite impact in mainland South-East Asia

**DOI:** 10.1186/s40645-024-00660-9

**Published:** 2024-11-20

**Authors:** Paul A. Carling, Toshihiro Tada, Ryuji Tada, Wickanet Songtham, Alan J. Cresswell, David C. W. Sanderson, Naomi Porat, Jaroon Duangkrayom, Stephen E. Darby, Praphas Chansom

**Affiliations:** 1 School of Geography & Environmental Science, University of Southampton, Southampton, SO19 1BJ UK; 2 State Key Laboratory of Geohazard Prevention and Geoenvironment Protection, Chengdu University of Technology, Chengdu, 610059 Sichuan China; 3 Institute for Geo-Cosmology, Chiba Institute of Technology, 2-17-1 Tsudanuma, Narashino, Chiba 275-0016 Japan; 4 Research Center for Earth System Science, Yunnan University, Chenggong District, Kunming, 650500 People’s Republic of China; 5 Northeastern Research Institute of Petrified Wood and Mineral Resources, Nakhon Ratchasima Rajabhat University, Nakhon Ratchasima, 30000 Thailand; 6 Environmental Physics, Scottish Enterprise Technology Park, Scottish Universities Environmental Research Centre, Rankine Avenue, East Kilbride, G75 OQF UK; 7 Geochemistry and Environmental Geology, Geological Survey of Israel, 32 Yesha’ayahu Leibowitz St, 9692100 Jerusalem, Israel

**Keywords:** Australasian strewn field, Tektite, Meteorite impact, Cosmic impact, Ejecta, Mainland South-East Asia

## Abstract

Aspects of the Quaternary sedimentary geology of South-East Asia have proven problematic in terms of interpretation as to the origins and relationships of the surface sediment layers. The MIS 20 large meteorite impact (*c*., 788 to 785 ka) occurred within mainland South-East Asia, evident from the well-researched ‘Australasian Tektite Strewn Field’ which extends over at least one tenth of the surface of the Earth. Key questions include: 1) whether the sedimentary impact signature is preserved in the Quaternary sediment cover of the region and 2) whether stratigraphic indicators and dating methods can discriminate meteorite impact-related associations of sedimentary strata, despite subsequent reworking and diagenesis. The importance of the questions raised relate to the search for the impact site, which has not been located conclusively. Moreover, the sedimentary signatures of meteorite impacts are not well known and the descriptions in this study should aid the recognition of impact signatures elsewhere in the world. An hypothesis was developed: Surface Quaternary sediments across a wide area of mainland South-East Asia represent the effects of a regionally significant meteorite impact. Over one hundred sedimentary sections were logged across five countries in mainland South-East Asia. Methods used, defining the stratigraphy and sedimentology, include computed tomography and X-ray scanning, geochemistry, magnetic susceptibility, and environmental luminescence as well as conventional grain size analyses. Luminescence analyses were applied to samples from key strata to provide age constraints and indications of reworking through dose distributional analysis of quartz fractions. The results of the investigation explain the nature of the stratigraphy and relate it specifically to the meteorite impact. In this manner, the strata and sedimentary signatures of the ejecta from a large cosmic impact are defined across a broad region, rather than being described at singular and isolated sections. The novelty is the spatial scale of the investigation which nevertheless remains detailed. A summary model of impact stratigraphy is presented that applies to the regional ejecta blanket covering at least 300,000 km^2^. Tektites were co-deposited with the ejecta and not introduced by surface processes reworking the deposits. Similar models may be applicable outside of mainland South-East Asia, wherever other large impacts are suspected to have occurred.

**Supplementary Information:**

The online version contains supplementary material available at 10.1186/s40645-024-00660-9.

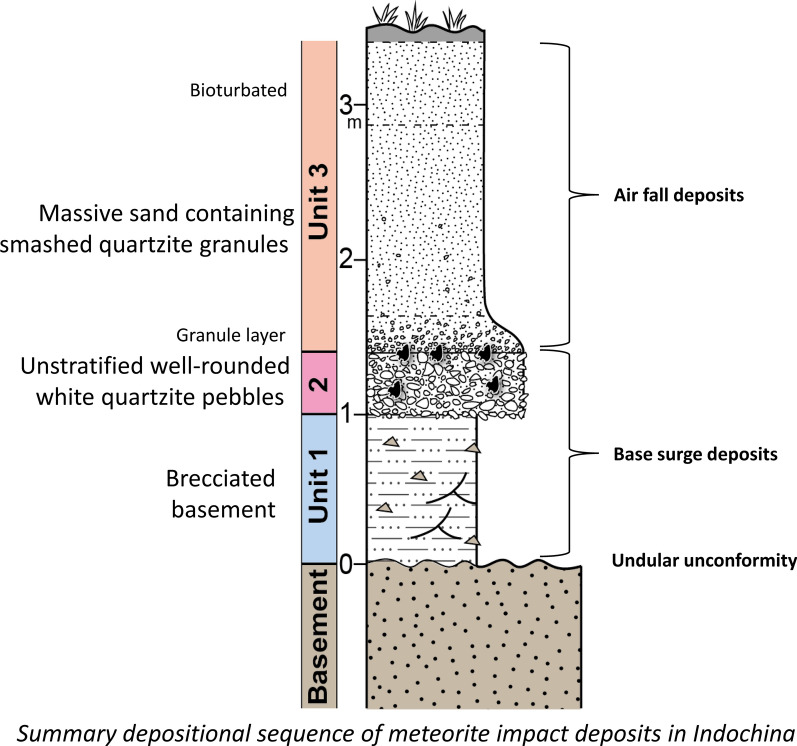

## Introduction

**Fig. 1:**
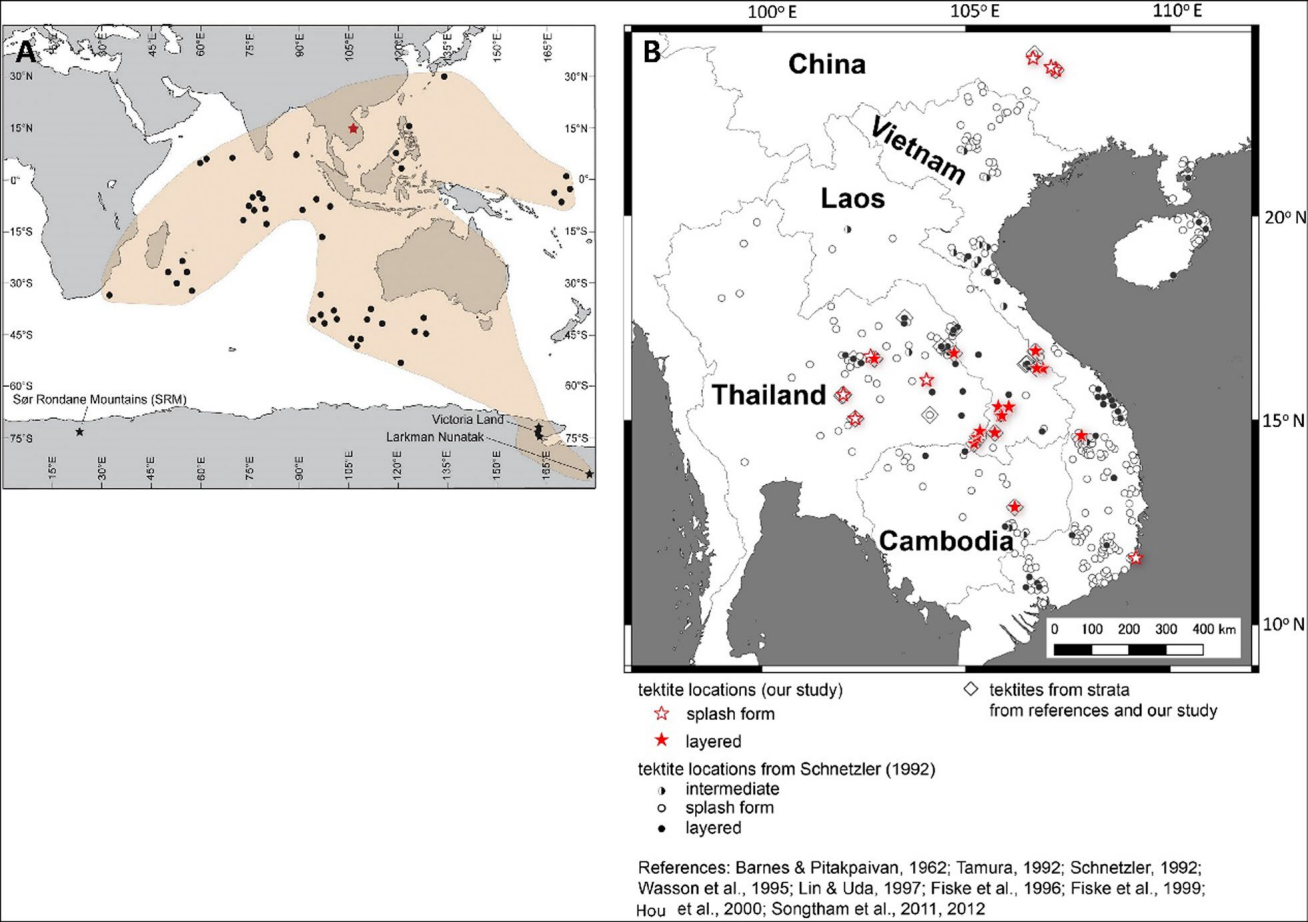
**A** Distribution of oceanic finds of microtektites that define the oceanic geographical spread of the ATSF (redrawn after Soens et al. 2021). NB: this panel does not include continental recovery sites, except for those locations reported by Soens et al. (2021) in Antarctica. The red star indicates the likely location of the meteorite impact as proposed by Soens et al. (2021 (see main text); **B** distribution of tektites across mainland South-East mainland Asia

A mid-Pleistocene (MIS 20/19) cosmic impact is believed to be responsible for the Australasian Tektite Strewn Field (ATSF) (Fig. 1) that extends over 10 to 15% of the surface of the Earth (Koeberl 1986; McCall 2001; Folco et al. 2023). The ATSF event likely occurred early within the transition of the Brunhes–Matuyama geomagnetic reversal (De Menocal et al. 1990; Schneider et al. 1992; Glass et al. 2004; Valet et al. 2014; Singer et al., 2019; Jourdan et al. 2019; Sieh et al. 2023). Despite considerable effort, the impact site has not been located conclusively. Various sites suggested have been reviewed by Koeberl (1994), Lee & Wei (2000), McCall (2001) and Glass (2003), amongst others, who concluded that a location within mainland South-East Asia between northeastern Thailand and southwestern Laos (Schnetzler 1992) or off the coast of Vietnam was most likely (Schnetzler et al. 1988; Schnetzler & McHone 1996; Seydoux-Guillaume et al. 2024).

Based on the distribution of the thickness of microtektite layers in marine sediment cores, the diameter of the crater associated with the impact has been estimated to be 32 to 116 km (Glass & Pizzuto 1994; Lee & Wei 2000; Glass and Koeberl 2006). The absence of an identified terrestrial impact site (Stauffer 1978) has been related to the possibility that: the local geology did not record the impact; the impact site is obscured offshore (Whymark 2021); or, if inland in China, the crater may be buried by sand dunes (Mizera et al. 2016; 2020); or multiple, small, geographically dispersed impactors did not leave a clear signature (Ford 1988). Wasson (1995) suggested a bolide produced a large aerial burst without impact, but the results of Fiske et al. (1996) indicate a large impact crater is likely (Glass & Pizzuto 1994; Cavosie and Koeberl, 2019). Although alternative locations have been proposed (Hartung & Koeberl 1994; Ma et al. 2004, 2004, 2004; Whymark 2021; Mizera et al. 2016, 2022; Karimi et al. 2023), Sieh et al., (2020, 2023) argue cogently that the impact crater is obscured beneath young lavas of the Bolaven volcanic field in Southern Laos, although this conclusion has been challenged (Mizera 2022; Mizera & Strunga 2024). For simplicity, herein we refer to a meteorite impact without prejudicing the nature of the event. The primary objective of this paper is to describe the sedimentary ejecta layers associated with the ATSF across mainland South-East Asia. In addition, the distribution of ejecta can assist in locating the impact site (Glass & Pizzuto 1994; Lee & Wei 2000; Glass & Koeberl, 2006; Morgan et al. 2006; Prasad et al. 2007; Glass & Simonson 2013), although determining the absolute extent of the sediment deposits is not addressed in this paper.

**Fig. 2:**
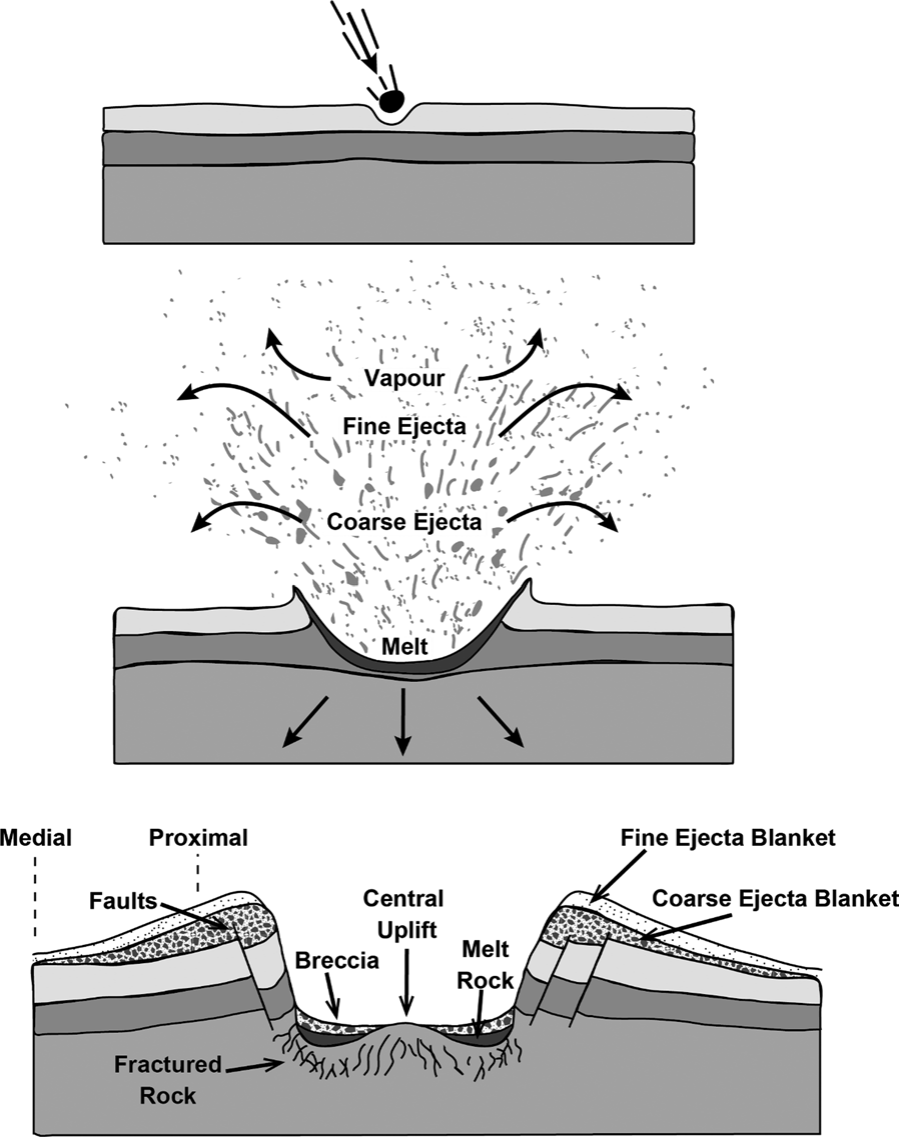
Schematic representation demonstrating disposition of coarse and fine ejecta blankets along a proximal to medial transect of an impact crater. Modified from unpublished illustration by Nelson 2018, http://www.tulane.edu/~sanelson/Natural_Disasters/impacts.htm

In a spatial context, the geological effects of a large impact might be considered as: (i) Proximal impact structures and proximal ejecta; (ii) Medial coarser ejecta plume fall-out and basal surge flow; and (iii) Distal ejecta plume fall-out, including tektites (Fig. 2) (see also French 1998; Stöffler & Grieve 2007 for more detailed considerations). Earth material is ejected beyond the rim of the crater due to shock and rarefaction wave energy in the form of a near surface ejecta curtain that is deposited as proximal and medial ejecta blankets (French 1998). Other ejecta is dispersed higher into the atmosphere along ballistic trajectories and tends to fall-out after the proximal and medial material is deposited and can contribute to deposits distal from the impact site. In addition, a ground-hugging base surge would have occurred (Knauth et al. 2005; Quintana et al. 2018; Schultz and Quintana 2017) that would have consisted of material released by the impact but also the surge would have eroded and entrained the weathered surface material surrounding the crater (Barlow et al. 2014), latterly depositing this material as a component of the medial ejecta deposits, before any high-lofted ballistic ejecta were deposited.

The majority of published impact studies have focussed upon the proximal geological effects of high-energy impactors (*i.e.* structural changes and the presence of impact lithologies; *e.g.* suevitic breccias and impact melt breccias; *e.g.* Kenkmann et al. 2014; Arp et al. 2019), rather than the medial and distal perturbations to the environment due to ground-hugging surge flow, the coarse ejecta trajectories, the consequent blanket layers (Oberbeck 1975; Amor et al. 2008, 2019; Branney & Brown 2011; Osinski et al. 2013), and the subsequent fall-out of fine sediment that initially had been entrained into the atmosphere by the impact (Koeberl 2007; Evans et al. 2008; Osinski et al. 2013). Although the distal ejecta of the ATSF has been recognized as a layer of microtektites (particle diameter < 1 mm) in marine sediment cores at multiple locations (*e.g.* Prasad et al. 2007), only recently have the coarse ejecta and the finer air-fall deposits been identified with any certainty on land within mainland South-East Asia (Tada et al. 2022; Sieh et al. 2023).

The primary objective, noted above, is accomplished by examining multiple locations in southern China, southern Laos, central and southern Vietnam, northern Cambodia, and north east Thailand where the relevant strata are exposed in section. Key questions include: 1) whether the sedimentary impact signature is recognizable and preserved in the Quaternary sediment cover of the region and 2) whether stratigraphic indicators and dating methods can discriminate meteorite impact-related associations of sedimentary strata, despite subsequent reworking and diagenesis. After preliminary examination of sections, a hypothesis was developed:

*“Surface Quaternary sediments across a wide area of mainland South-East Asia represent the regionally extensive effects of a meteorite impact.”*

The aim of the study is to better understand the nature of the sedimentary signature associated with the impact event and to provide a definitive regional sedimentological study that might assist in identifying other impact sites worldwide. Presently, the only direct association in the scientific literature between the impact event and the surface sedimentary sequences in the region has been presented by Tada et al. (2022) and Sieh et al., (2023). However, large rafts of ‘burnt?’ trees in river terraces in north east Thailand (Bunopas et al. 1999; Howard et al. 2003; Haines et al. 2004; Sangsuk 2008) have been suggested as being due to the impact, and some sandy deposits on the land surface have been related tentatively to the impact (Bunopas 1990, 2021; Bunopas et al. 2003).

### Existing sedimentary evidence for meteorite impact

The Australasian tektite strewn field is distributed widely south of a northern limit running between Maoming, Guangdong Province, to Baise in the Bose basin, western Guangxi Province (Yuan et al. 1999; Hou et al. 2000; Lee & Wei 2000; McCall 2001). As detailed below, the main recorded concentration of tektites lies within mainland South-East Asia (Fig. 1), but the spread extends to Australia and Antarctica (Folco et al. 2016; Di Vincenzo et al. 2021). Microtektites and a few tektites have been recovered from marine sediment cores in the Indian Ocean, Philippine, Sulu and Celebes Seas, South China Sea, Sea of Japan, and the western Pacific Ocean (Koeberl 1986; Schneider et al. 1992; Schnetzler 1992; Ma et al. 2004; Glass and Koeberl 2006; Prasad et al. 2007). The estimated total mass of tektites ranges from 1 × 10^11^ to 3 × 10^13^ kg (Schmidt et al. 1993).

Tektites occur widely and frequently in north east Thailand (Songtham et al. 2011). All examples are black and glassy, either layered or splash form (Fig. 1). The tektites are derived from crust material of the Earth (Koeberl 1986, 1992) and layered forms—Muong Nong type (Lacroix 1935)—may incorporate relicts of incomplete melting of a single, fine-grained sedimentary formation (Blum et al. 1993; Glass and Koeberl 2006) that solidified shortly after hyper-velocity impact and indicate proximity to the impact location (Blum et al. 1993; Ma et al. 2004; Rochette et al. 2018). In contrast, splash-form tektites indicate liquid or plastic melt followed by melt solidification outside of the atmosphere (Mizote et al. 2003; Žák et al. 2019; Gattacceca et al. 2022), farther away from the impact site, and show ‘aerodynamic’ shape, homogeneous texture, and low volatile content (Koeberl 1992; Schnetzler 1992).

Fiske et al. (1999) recovered large quantities of large, layered tektites in central Laos and determined that the layered tektite distribution extended over > 50,000 km^2^ across central Laos, central Vietnam and north east Thailand. In contrast, Sieh et al (2023) estimated that the area of distribution was ~ 125,000 km^2^. Numerous layered tektites have been recovered by amateurs from unrecorded ground surface locations across north east Thailand, but plentiful examples also have been recorded within near-surface sediment sections (Howard 2011), usually within a gravel layer that frequently underlies the regionally distributed Yasothon sandy ‘soil’ series (Barnes & Pitakpaivan 1962; Chongprai & Chotimon 1971; Yabuki et al. 1981; Satarugsa 1987; Löffler & Kubiniok 1991; Parry 1992; Tamura 1992; Wasson 1995; Fiske et al. 1996, 1999; Lin & Uda 1997; Haines et al. 2004; Nichol & Nichol 2015; Mizera 2022; Songtham et al. 2011, 2012; Duangkrayom et al. 2022). Microtektites also have been recovered from the surficial terrestrial sediments (Bunopas et al. 1999), and Howard et al. (2000) tentatively reported shocked quartz in a layer immediately above a tektite-bearing horizon in north east Thailand, as have Tada et al. (2022). However, the depositional age of the host sediments usually is considered to be younger than the formation age of the tektites (*e.g.* McCall 2001). Thus, it has long been debated whether the tektites are deposited in situ or reworked (Fiske et al. 1996; Koeberl & Glass 2000; Howard 2011; Langbroek 2015). Considering the possibility of reworking of tektites, the presence of tektites within any stratum usually is insufficient to identify the ejecta layer (Fiske et al. 1996; Koeberl & Glass 2000), an issue addressed latterly in this study.

Over several decades, a Pleistocene age for the tektites has been determined by numerous laboratories using K–Ar, Ar–Ar, and fission track dating and is conventionally placed at about 800 ka (Lowman 1964; Chalmers et al. 1976; Yamei et al. 2000; McCall 2001; Haines et al. 2004; Howard 2011; Hou et al. 2000), *i.e.* before the Brunhes–Matuyama reversal, *c*., 781 ka. Some early attempts at dating tektites placed the event after the reversal Gentner et al. 1969; Gartner et al. 1969; Fleischer et al. 1969; Tamura 1992; Ky 1989; Guo et al. 1996). However, thermal resetting of the fission track clock by wild fires may result in ages younger than the true formation age (Westgate et al. 2021). Recently, precise dating by Jourdan et al. (2019) provided a weighted mean age of 788.1 ± 2.8 ka (± 3.0 ka, including all uncertainties) comparable with the 785 ± 7 ka age reported by Schwarz et al. (2016). Thus, the uncertainty in the age of the tektites is now greatly reduced such that they can be associated with a large meteorite impact shortly before or during the Brunhes–Matuyama reversal.

### Enigmatic regional Quaternary geology

Within a regional context and considering only those deposits relevant to the impact story, unlithified Holocene and Pleistocene surface sedimentary deposits have only been well described in the region of the Khorat plateau, north east Thailand, and southern China. Yet, as is explained below, there is no consensus as to interpretation. For brevity, a description is supplied below for north east Thailand that reasonably applies to sections investigated during this study across mainland South-East Asia. Where significant deviations from this description are relevant, these are considered in Results and Discussion.

**Fig. 3:**
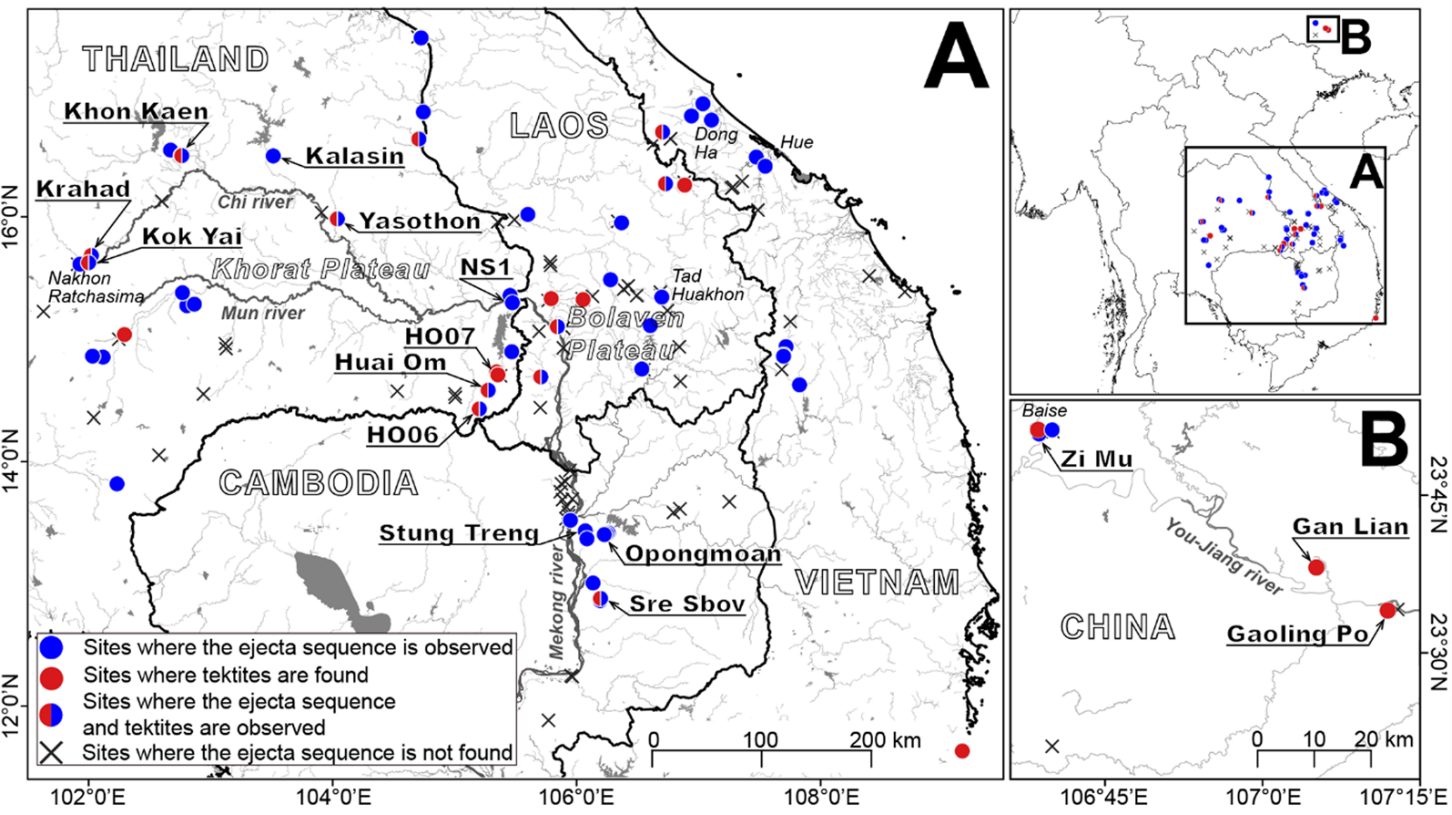
Locations where sedimentary sections were examined during this project. **A** Main study area; **B** Study area in southern China. See Table S2 and Fig. S1

Herein the term ‘basement’ is used to denote that rock formation that lies immediately below the surface sedimentary deposits that are the focus of this study. In north east Thailand, the basement largely consists of the Khorat Group (Mesozoic; Racey et al. 1996): claystone, siltstone, sandstone, and evaporites that have weathered to a limonitic sandy clay (Ridd et al. 2011). Petrified ‘fossil’ wood is abundant in the basement and occurs also as reworked material in overlying deposits (Boonchai et al. 2017; Mustoe et al. 2022). These Mesozoic sedimentary rocks extend into southern Laos and northern Cambodia as Jurassic-Cretaceous sandstones (Racey 1996) interbedded with small amounts of mudstones (Bárdossy & Aleva 1990; Vilayhack et al. 2008). In the south of north east Thailand, Neogene basalt and andesite occur locally and more extensively in the vicinity of the Bolaven Plateau in southern Laos. It has been suggested that some basalt flows may have been generated by the impact event (Ford 1988), although it should be noted that basalt formation was widespread across mainland South-East Asia during the Pleistocene (Fedorov & Koloskov 2005) with the youngest basalt ^40^Ar/^39^Ar dated to 27 ka ± 11 ka (Sieh et al. 2020). Many sedimentary sequences display lateritic tendencies in the topmost few metres below the land surface. We use a general terminology whereby 'laterite' includes plinthite (lateritic soil). Laterite is geochemically characterized by the depletion of Si and enrichment of Fe and Al. Above the basement, Fe-oxide cemented gravel beds, from < 1 m to several metres thick, occur arcing around the courses of the Mun river and the Chi River; these rivers are shown in Fig. 3, although, traditionally regarded as river terrace deposits (Moormann et al. 1964; Udomchoke 1989; Parry 1992; Dheeradilok 1993), Michael (1981) and Yoshiki (2000) argued against the presence of terraces per se. Yoshiki (2000) makes an argument that the gravels are lag-concentrated exposures of the uppermost formation within the Khorat Group, which contains similar conglomerates. Regardless, these gravels are locally openwork, lack a distinct orientated fabric, are very poorly sorted and largely unstratified (rarely cross-stratified; Löffler & Kubiniok 1991), and traditionally were viewed as braided river deposits. Importantly, the upper *c.,* 1 m thickness appears to be freshly reworked (in contrast to the lower gravels), and Carbonnel and Dulaix (1971) and Parry (1992) suggested that these upper thin deposits were high-energy water flow or debris flow deposits. However, thin (< 1 m), wispy, gravel beds also occur extensively at the base of a cover sand well away from the main watercourses, including on the low-gradient or flat Khorat Plateau, where traditionally they have been regarded as a weathering residuum (Yoshiki 2000). The low surface gradients on the plateau would not be conducive to widespread energetic water flows of regional extent, and Löffler & Kubiniok (1991) had difficulty accepting fluvial processes as the origin for the gravels. Similar gravel beds extend into southern China (Wang et al. 2018) as well as more widely in Cambodia, Laos, and Vietnam. In terms of lithology, clasts are usually well-rounded quartz, quartzite, and rounded and platy sandstone with some flint and chert, mostly derived locally (Choowong 2011) or from more distant but regional outcrops (Tamura 1992). The sandstone clasts are sometimes weathered, but there is little evidence of significant weathering of quartzite clasts, many of which are fractured. The top of the gravel beds is usually lateritic, commonly < 1.5 m thick (Kheoruenromne 1987; Tada et al. 2022). The contact between the gravels and the cover sand is usually distinct and often highly undulating (Moormann et al. 1964; Parry 1992) although Wang et al. (2018) have argued that the fine matrix of the gravel in southern China is infiltrated downwards from the cover sand.

The cover sand, up to several metres thick, is widely reported throughout southern China, Laos, Vietnam, northern Cambodia, north east Thailand (Hoang Ngoc 1989; 1994; Demeter et al. 2010; Liu & Deng 2014; Wang et al. 2018) as well as further afield, blanketing the landscape irrespective of the local topographic gradients (Löffler et al. 1984). This distinctly red sand (*e.g.* Munsell colour: 2.5YR 5/3; 10R 5/8) can be 3 m to 15 m thick in undisturbed locations but frequently is truncated by agriculture. The unit can lie directly upon the weathered, brecciated, or lithified basement rocks or in sharp contact with the gravel deposits described above. One or more thin plinthite layers (< 0.20 m thick) can occur high in the sandy sediments, as loose, pisolithic collections of concretion nodules (Dudal & Moormann 1964; Parry 1992). Visually, the sand is massive and is composed primarily of well-sorted silt to coarse sand, sometimes with more clay (10 to 20%) lower in the profile and scattered pisolites and distinctive shattered white quartz granules throughout. This virtually mono-mineralic quartz sand (Supplementary Table S1) is capped on higher ground by typic kandiustults (USDA 1999)—the Yasothon, Khorat and Warin soil series that are all very similar in character—otherwise variously described as rhodic ferralsols (Moormann et al. 1964), oxic paleustults (Kheoruenromne 1987), or rhodostults (Yoshinaga et al. 1989), that originally may have been much more extensive but have been eroded and removed (along with the cover sand) at lower elevations (Moormann et al. 1964). Here for brevity, these soils are referred to collectively as the Yasothon series. The Yasothon A-horizon is between 10 and 30 cm thick, and the B-horizon extends only to a depth of 0.80 to 1.2 m (Land Development Department, 2004) at which occasionally a thin, biogenic stone line occurs.

The origin of the cover sand is enigmatic having been related tentatively to lacustrine (Dheeradilok & Chaimanee 1986; Dheeradilok 1987); marine (Nguyen 1994); colluvial (Michael 1981); fluvial (De Dapper 1987; Hokjaroen & Parry 1989; Nutalaya et al. 1989), or aeolian (loess-forming) processes (Boosener 1977, 1987, 1991); Boonsener & Tassanasorn 1983; Löffler et al. 1983; Sonsuk & Hastings 1984; Udomchoke 1989; Ky 1989; Parry 1992; Šibrava 1993; Wang et al. 2018); the latter due to a supposed regional arid climate during the last glacial period (Löffler et al. 1984). However, Prone et al. (2005) and Wang et al. (2018) suggested a distal aeolian origin with the sand originating in central China and commented on grain surface features that indicated very high-energy impact between grains and crushing traces, as have Om et al., (2023) more recently. The regional aeolian interpretation is based solely on the supposedly homogeneous, well-sorted texture, and sub-spherical sub-rounded grain shapes (Tamura 1992; Nichol & Nichol 2015), but the regional aeolian (loess) explanation is difficult to reconcile with the relative coarseness of the deposits and the presence of extensive rain forest during the Middle to Late Pleistocene (Phillippe et al*.*, 2013; Raes et al. 2014). The vegetation associations during the Holocene in north east Thailand are less clear (Penny et al. 1996; Kealhofer & Penny 1998), but significant aridity is not indicated during the Pleistocene. The fact that fine well-sorted quartz is not concentrated high in the profile, as might be expected for an aeolian cover sand (Yoshinaga et al. 1989), is an issue. However, red cover sands in southern China, similar to the Yasothon series, have been attributed to aeolian deposition *c*., 800 ka, due to strengthening of the East Asian monsoon at that time bringing sediment from both regional and distal sources (Hong et al. 2013; Liu & Deng 2014). In contrast, early soil studies (see Moormann et al. 1964) as well as more recent investigations (Löffler & Kubiniok 1991; Xi 1991; Huang et al. 1996; Zhu et al. 2004; Choowong 2011) proposed that the cover sand is a Late Tertiary weathering mantle formed under humid tropical conditions, latterly bioturbated primarily by termites (Moormann et al. 1964; Löffler & Kubiniok 1991; Sanderson et al. 2001; Om et al. 2023). Bunopas (1990) proposed that the cover sand was a ‘loess’, deposited catastrophically from ejecta dust fall due to the Pleistocene meteorite impact (*i.e.* the cover sand is *c.,* 800 ka old if associated with dated tektites) and he termed these deposits ‘catastroloess’.

The cover sand is not securely dated (Songtham et al. 2015). Although Parry (1992) averred a mid-Pleistocene age for the sand, samples from separate studies of the top 2.5 m thickness have produced C^14^ dates of 8190 a BP and 6620 a BP (Sonsuk & Hastings 1984; Udomchoke 1989, respectively), whilst Sanderson et al. (2001) using luminescence dating also concluded the sands were Holocene for a similar depth range as for the C^14^ assays, with older late Pleistocene material beneath. Punpate et al., (2005) using luminescence dating of two samples roughly 40 to 50 cm below the surface provided dates of 25.9 ± 4.3 ka to 28.9 ± 6.3 ka, respectively. Samples at these shallow depths are subject to bioturbation (Supplementary Information) which may account for the differences in the dates obtained in different studies. In locations close to the major rivers, the underlying gravel may represent mid-Pleistocene river deposits that Tamura (1992) considered to pre-date the meteorite impact, or gravel layers within the basement (Yoshiki 2000). However, the thin wispy gravel deposits that occur widely across the Khorat Plateau above a sandy brecciated layer and below the Yasothon series remain undated. Parry (1992) suggested that all the gravels were Mid-Pleistocene in age.

To test the hypothesis posed above, over 140 sedimentary sections distributed across mainland South-East Asia (Fig. 3 & Supplementary Table S2) were examined and, for selected locations, a range of methods was applied to study the detrital composition. We did not sample river terraces (Howard et al. 2003; Haines et al. 2004) that contain the remains of Quaternary trees (Mustoe et al. 2022).

## Construction and content

Brief notes were made of all sections, and, for most, the stratigraphy was photographed, measured, and sketched at the outcrops recorded within Fig. 3. For several key sections, sediment samples for grain size, bulk density, environmental luminescence, and magnetic susceptibility profiling were collected down vertical profiles by driving rigid plastic or copper canisters (3 cm^3^) into cleaned sections, usually at spacings of 10 cm. The purpose of these measurements was to define the vertical variation in the sedimentology of the deposits. Environmental luminescence and magnetic susceptibility are particularly useful to define cryptostratigraphy within fine sediments similar to loess (*e.g*. Staff et al. 2024; Bradák et al. 2021). Samples for grain size analysis were dry sieved to > 1 mm. Particle size distributions < 1 mm were obtained from the average of five replicate analyses using a Malvern Mastersizer 3000 using standard procedures (Blott et al. 2004), and particle densities and bulk densities of block samples were determined as weight per volume of sample. Elemental components of the cover sand were determined primarily using X-ray fluorescence (XRF: see Supplementary Information: Micro-XRF analysis and Table S2), although scanning electron microscopy (SEM) was used on some samples, and mineral composition was determined using X-ray diffraction (XRD). Infrared-stimulated luminescence (IRSL) and blue light-stimulated luminescence (BLSL) integrated optically stimulated luminescence (OSL) intensities, depletion rates, and IRSL/OSL ratios were used to develop relative age profiles (Sanderson & Murphy 2010; Muñoz-Salinas et al. 2014; Portenga & Bishop 2015) for selected sections using a Scottish Universities Environmental Research Centre (SUERC) portable OSL reader (Sanderson & Murphy 2010) with measurements conducted on bulk materials for samples collected in 2018 and for separated < 500-µm polymineral grains for samples collected in 2019. The luminescence analysis focussed on the cover sand, although for one section the analysis was extended into stratigraphic units below the cover sand (further details are contained within Results).

Large tektites were recovered in situ from inspection of outcrops, and ex situ examples were also recorded when found on the floor of excavations close to recorded sections. Fourteen samples for OSL and thermally transferred (TT-OSL) luminescence dating were obtained using 6.5 cm internal diameter, 10 cm long (33 cm^3^) aluminium tubes driven into the sections at key locations. For bleach control, samples of sediment adjacent to the luminescence sampling sites were dried and dispersed on a large tray in the field for 2 h, stirring occasionally to ensure full exposure to bright sunlight. OSL, TT-OSL, and other quartz deep trap analyses were conducted at SUERC (Cresswell et al. 2018a, b, 2019a, b, 2022) and at the Geological Survey of Israel, Jerusalem. Full details of OSL and TT-OSL profiling, and OSL dating procedures are given in Cresswell et al. (2018a, b, 2019a, b, 2022) and Faershtein et al. (2020). Magnetic susceptibility samples were prepared (after Walden et al. 1999) for low-field (LF) and high-field (HF) susceptibility measurements using a Bartington MS2B single-sample dual-frequency sensor. Net luminescence intensities (OSL and IRSL) measured up through the sedimentary sections provide some indication of variation in the sensitivity and dosimetry within the stratified sequences. For example, the sensitivity has a high capacity for discrimination of concentrations of quartz sediment grains (Sawakuchi et al. 2020) and is a useful parameter to pick-out stratigraphic changes where none are visible (Sanderson et al. 2001, 2010). Similarly, OSL Front considers the initial faster bleaching portion of the decaying signal. The variation in the depletion rates of the signals may reflect changes in colour or diagenetic properties of deposits, whereas the signal ratios may indicate mineralogical variations.

Two rectangular intact sediment blocks were cut from the base of a cover sand section near Krahad, Noen Sa-nga, Chaiyaphum, Thailand (see Table S2 for location), using a spade and trowel. In the laboratory, these blocks were pared down using a scalpel to form slabs 300 mm high, by 200 mm wide and 10 mm thick. The two slabs were subject to X-ray analysis although one cracked before X-ray and this crack is clearly seen on the images. The slab which did not crack was subject to computer tomography (CT) scanning at Suranaree University of Technology Hospital, Thailand.

## Results and interpretation

### Stratigraphy of key sections

**Fig. 4:**
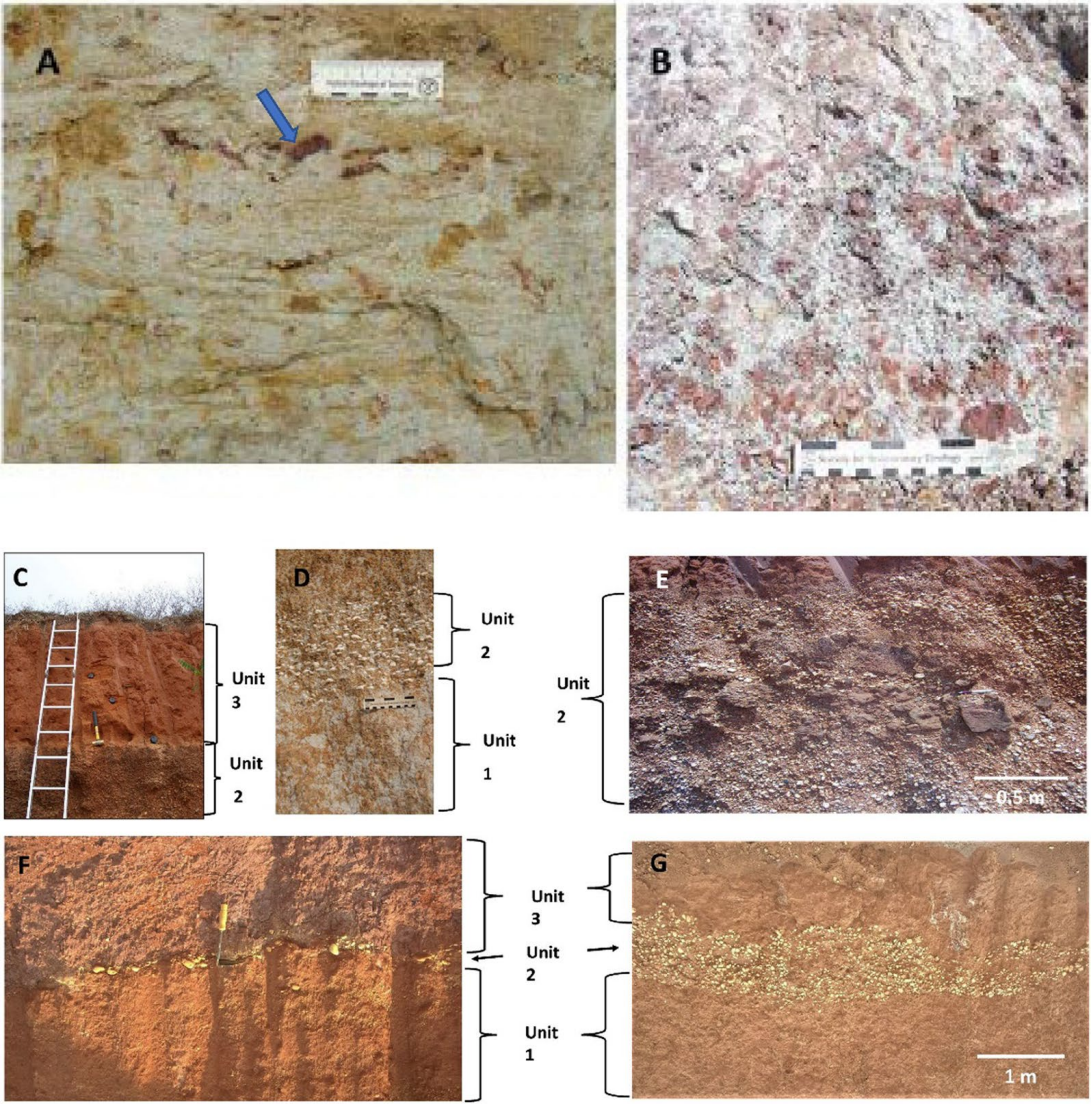
A selection of sections across the region showing typical sedimentology and stratigraphy. **A** Sandy phase of Unit 1 breccia, including several small dark red sandstone angular clasts (arrowed below scale bar); **B** Weathered basement sandstone below Unit 1. Scale bar in divisions of cm; **C** General view of cover sand (Unit 3) overlying gravel layer (Unit 2); **D** Close-up of breccia (Unit 1) below distinctive gravel layer here consisting of white pebbles (Unit 2); **E** Unit 2 containing poorly bedded white pebbles and laterite angular clasts: **F** Interface between Unit 1, a thin Unit 2 (white pebbles) and Unit 3; **G** Typical Unit 2 showing variable thickness over a short lateral distance but with well-defined lower and upper interfaces with Units 1 and 3

**Fig. 5:**
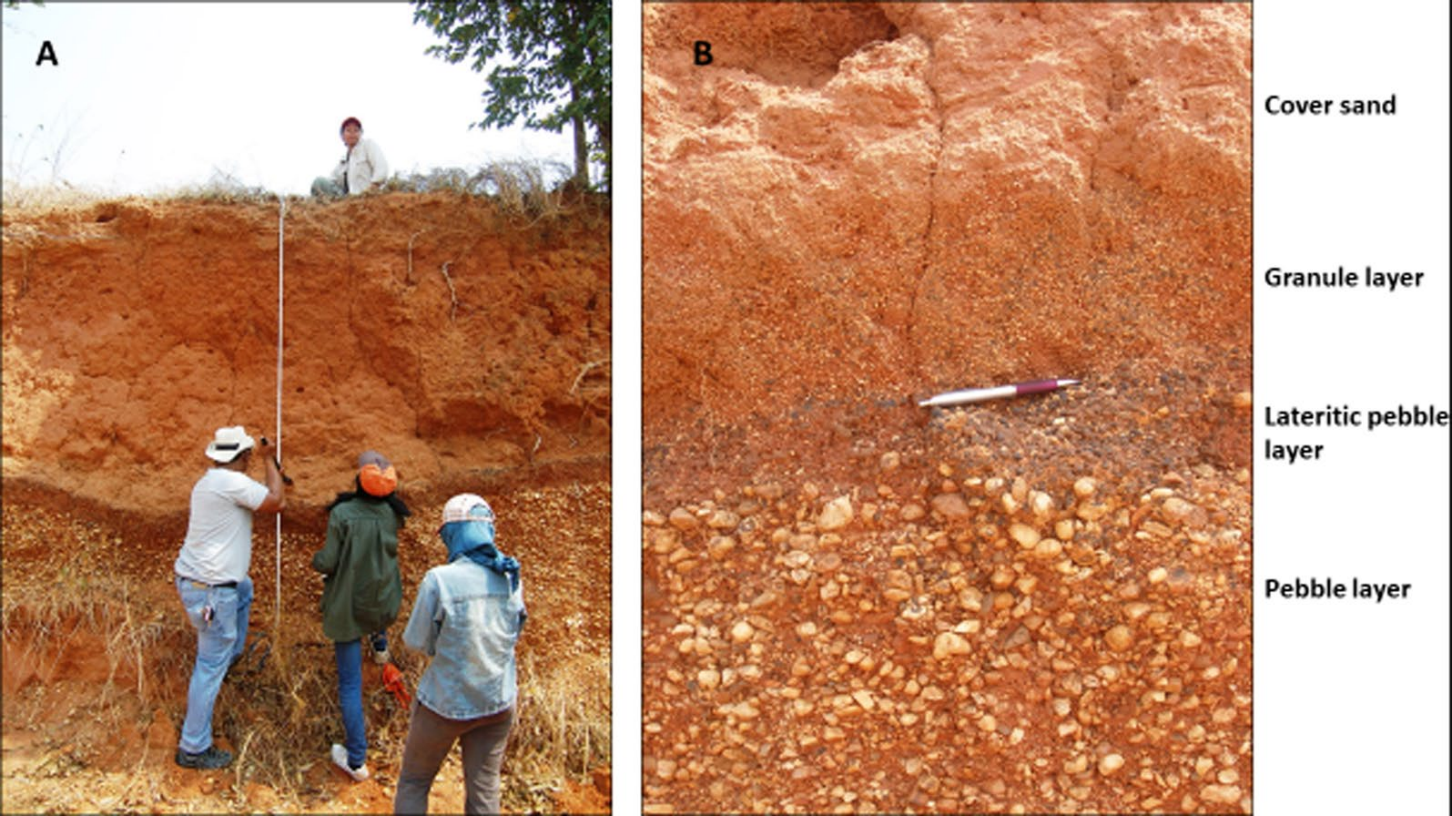
**A** General view of section at Kok Yai. A gravel layer (Unit 2—white pebbles behind figures) is lateritic at the top (dark hue) with cover sand (Unit 3) above. Unit 3 is commonly truncated by agriculture (surface below upper figure); **B** Close-up of interface between gravel layer (Unit 2—pebbles) that fines upwards to granules (lateritic dark hue). Dispersed granules are prominent (above the pen) at the base of the cover sand (Unit 3), typically with an overall thickness of 10 to 20 cm. The basement is not exposed in panel A, but at this location it consists of well-rounded small cobbles which appears to be a river gravel (see Fig. 7)

Four typical sections are described below selected from sites in north east Thailand. Similar sites occur in Laos, Vietnam, and Cambodia, the selected results from which will be published in due course. The GPS locations of all the sections are recorded within Supplementary Table S2 (see also Fig. 3). As is explained below, although the sedimentology may vary, at each locality we recognize the same basic stratigraphic succession: Basement or weathered basement; a ‘breccia’ that can be replaced by a sandy phase (Unit 1; Fig. 4); a gravel layer reworked from basement-derived clasts (Unit 2; Fig. 4) and an overlying cover sand (Unit 3; Fig. 5) that usually contains a granule layer (often diffuse) at its base. The cover sand, above the granule layer, is homogeneous to the eye but can be sub-divided, as is explained subsequently. Major undulations frequently occur at the interface between the basement and Unit 1 and between Unit 2 and 3, so a separate section considers this observation: ‘Undulating Interfaces between Basement and Unit 1, and between Units 2 and 3’.

#### Huai Om (Thailand)

14.57922° N; 105.275° E.

The main section described below is *c*., 90 m to the west of the section described by Fiske et al. (1996). This exposure is referred to as subsection B by Tada et al. (2022) wherein further details can be found. The section is 3.1 m high.

*Basement—weathered local rocks:* The basement at the section consists of Cretaceous cross-bedded sandstone (24 – 27^o^ ripple-laminated foresets) that is weathered (red mottling) in the upper 1 m thickness. A red mudstone lens was located on top of the sandstone 200 m to the east of the section. The sandstone basement was more extensively exposed nearby at HO7 (Table S2) where vertical jointing and the overlying red shale were evident. At location NS1 (N 15.2958; E 105.4738, 80 km to north of Huai Om), the basal sandstone is distinctly cross-bedded with concentrations of quartzite pebbles at the base of tabular foresets.

**Fig. 6:**
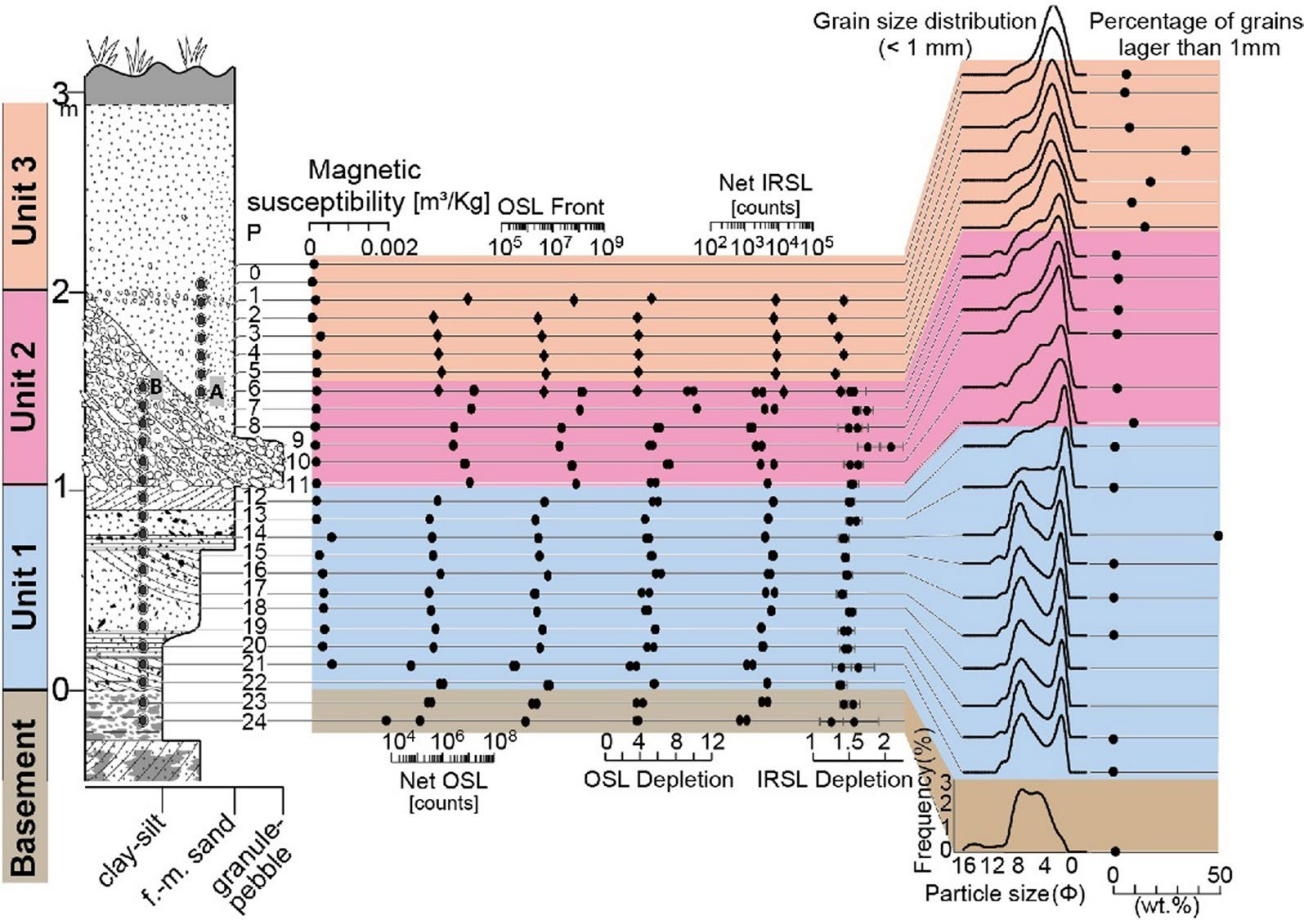
Summary stratigraphy for the Huai Om section. The point (P) numbers represent the samples mentioned in the text. Mass specific magnetic susceptibility (m^3^/Kg) and portable OSL and IRSL (counts) values highlight the cryptostratigraphy, with grain size curves on the right of the figure. Grain size distributions in Unit 1 mainly contain small quantities of fine gravel (except sample 14). Unit 2 consists of pebble gravel, but only the matrix grain size distribution is given here. NB: The interface between Units 2 and 3 slants downwards to the right between samples 6A and 6B. The small gravel content in Unit 3 is due to the presence of isolated granules, except for sample P4 which contains gravel-sized plinthite concretions. Portable OSL and IRSL measurements were conducted on bulk materials for 2018 samples (diamonds) and separated < 500 µm polymineral grains for 2019 samples (circles), with sample 6 common to both measurements

*Unit 1: Breccia:* For brevity, the term breccia is used throughout this report in the sense that the deposits often consist of angular fragments of the basement rock bound by partially cemented matrix of small particles. However, sandier cemented and weathered deposits can replace the breccia (as here) wherein angular particles are fewer. A 60 cm thick deposit (Unit 1 in Fig. 6) of soft, grey-yellow clay-rich fine sand that coarsens upwards from fine to medium sand and then to fine sand and clay, with ill-defined thin laminations, lies above a distinct interface with the basement. The sediment grain size distributions are strongly bimodal (Fig. 6). Red imbricated siltstone chips (*c*., 1 cm) occur along the interface with the basement, and unoriented chips occur again towards the top of the unit along with a few white (clay?) chips. Smaller (< 1 cm) siltstone chips occur throughout, and a few angular small sandstone and sub-rounded quartz pebbles occur scattered within the lower profile. Grey convolutions (10 to 20 cm thick) occur in a soft fine sand bed (sample P14), just below a firm bed (sample P13 in Fig. 6) of laminated red coarse sand containing some granules and a few small pebbles. The soft fine sand bed exhibits a gradational laminated wavy contact with the coarse sand bed above.

*Unit 2: Gravel:* Above the coarse sand bed is a gravel layer consisting mainly of rounded quartzite pebbles, although angular sandstone gravel and fractured rounded pebbles occur frequently. The bed varies rapidly in thickness over a *c*., 2 m distance from 1.0 m on the left of the exposure to 0.2 m on the right of the exposure and are locally cross-bedded. A gravel stringer (at P7; Fig. 6) extends to the right of the unit. Fragments of petrified wood also occur infrequently. The fabric of the gravel layer is indistinctly but continuously laminated with slight indication of coarsening upwards. This fabric differs from most other Unit 2 sections in the region wherein the gravel fabric is usually chaotic. The change in the thickness of the gravel layer from Huai Om to HO7 (17 km distance), where the gravel is reduced to a stringer, indicates the spatial variation in the gravel layer thickness, which variation was noted elsewhere in the region by Sieh et al., (2023). The similarity of the grain size distributions of the Unit 2 matrix (Fig. 6) with the size distributions of the overlying cover sand suggests that the material is of the same origin.

***Unit 3: Cover sand:*** The cover sand, here grey to reddish grey, has many scattered, rounded small quartzite pebbles and granules which become smaller and less frequent up the section except within the gravel stringer. Most of the quartzite fragments are highly angular and are dispersed throughout the finer matrix. The lower 110 cm thickness of cover sand is partially laterized but continues upwards as 40 cm of soft cover sand. A lateritic gravel stringer, at the interface of Units 2 and 3 (Fig. 6), is < 30 cm thick and consists of a high concentration of granules dispersed within the cover sand, above which the cover sand continues for a further c., 1 m as a soil, evident by bioturbation and an increased organic content. The grain size distributions are negatively skewed and fine slightly upwards (Fig. 6). The change in the luminescence parameter values between sample P2 and P1 indicates the presence of a stratal break, such that sample P1 represents the soil horizon (otherwise unsampled) overlying the lower distinct layer represented by samples P2 to P6 (Fig. 6).

Significantly, Tada et al., (2022) report shocked quartz from all three units and suggest that the Units 1–3 represent a sequence of ejecta deposits. Further consideration of the Huai Om section and Fig. 6 is given in section '3.3 Environmental Luminescence, Grain size and Magnetic Susceptibility Profiles*'*.

#### *Kok Yai (Ban Basieo)* (Thailand)

15.60922° N; 101.9846° E.

**Fig. 7:**
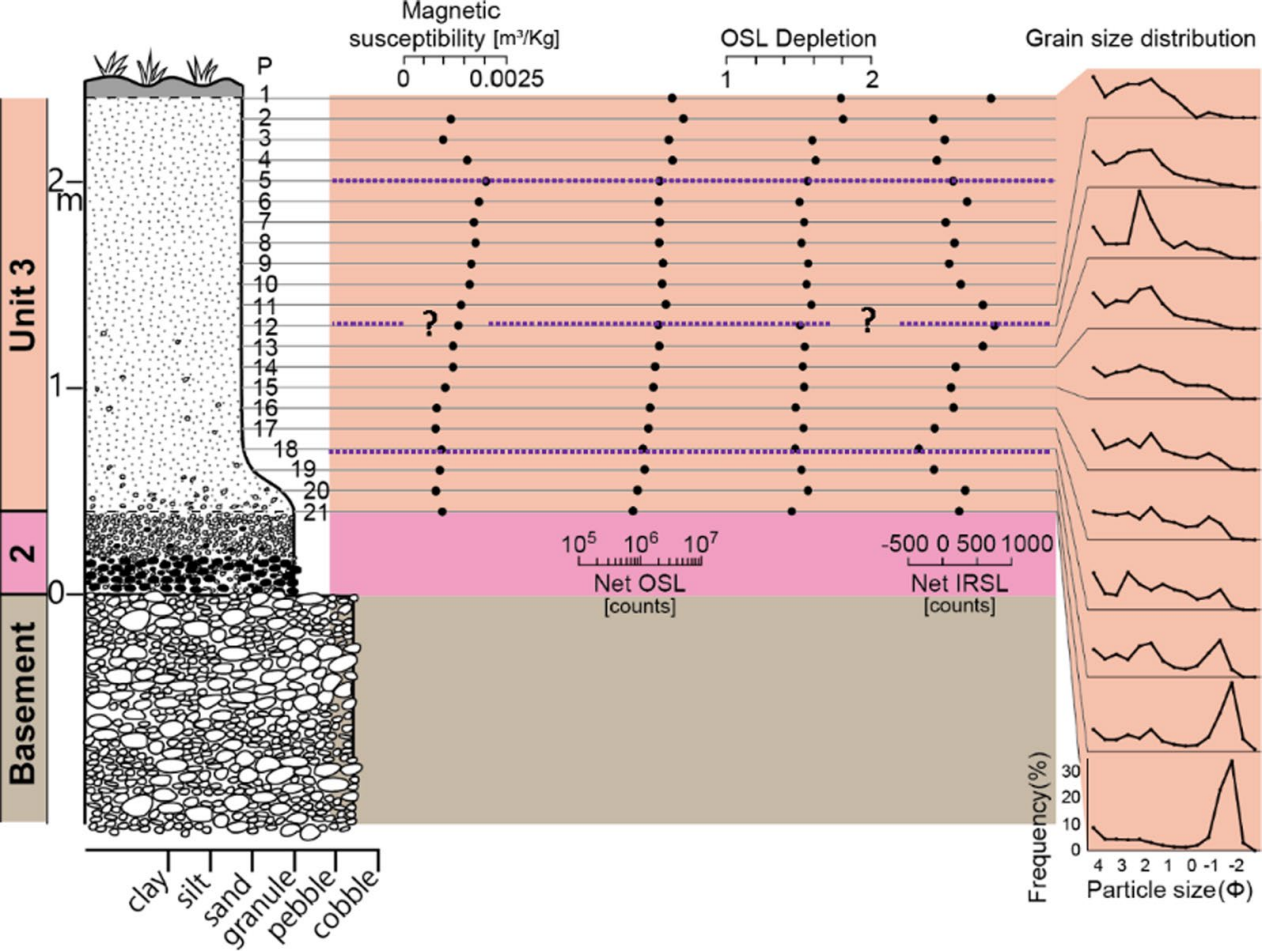
Summary stratigraphy for Kok Yai. Point (P) numbers represents the samples mentioned in the text. Mass specific magnetic susceptibility (m^3^/Kg) and portable OSL and IRSL (counts) values highlight the cryptostratigraphy, with grain size curves on the right of the figure. Sub-divisions of Unit 3 are shown as purple pecked lines

*Basement—weathered local rocks or widespread river gravels:* The lithified basement rocks of the Cretaceous Maha Sarakham Formation that crop out in the vicinity are widely distributed in the region (Racey et al. 1996), but this Formation is not exposed at this location. Rather the character of the basement is more consistent with the Khok Kruat Formation that lies immediately below the Maha Sarakham Formation (Racey et al. 1996), with > 10 m metres thickness exposed, consists of thinly bedded, white, well-rounded pebbles that are clearly weakly stratified (Fig. 7), often cross-bedded, reminiscent of braided river deposits (Songtham et al. 2012). These basal pebbles are unfractured in contrast with the Unit 2 described below. The upper surface of this unit undulates strongly.

*Unit 1:* Breccia is absent in contrast with Huai Om.

*Unit 2: Gravel:* A pebble gravel layer, up to 1.8 m thick (40 cm thick in Fig. 7), lies unconformably, usually along a distinctly wavy interface, above the basement fluvial pebbles deposits. Consisting mainly of well-rounded quartzite pebbles that are often broken, it is very well sorted with no evident pebble orientations. Broken rounded pebbles exhibit angular facets, but no quartzite pebbles were found that exhibited intact internal fractures. From this observation, it is concluded that high-impact forces broke the pebbles rather than weathering subsequent to deposition being the cause. The unit is largely unstratified, with occasional weak stratification that mainly fines (occasionally coarsens) upwards at this location. This gravel layer appears to be reworked from basement river gravels during a distinct transport event that resulted in the unconformity. The upper 20 to 30 cm thickness of this quartzite pebble bed can be laterized (although this is not shown in Fig. 7, it is evident in Fig. 5).

*Unit 3: Cover sand:* The base of the cover sand consists of a layer of granules (samples P21 to *c.* P19 in Fig. 7) that is integral to the cover sand lying above it is usually 10–20 cm thick on high points of a wavy contact between the reworked gravel layer and the cover sand, thickening from 0.5 m to 0.7 m in the intervening hollows. Here the granules are closely spaced to form a non-contact framework, although elsewhere near this section, and across the region, granules can be in contact or more diffusely spaced within a matrix of the cover sand. The granule layer is laterized in the hollows but not on the high points. Laterization can extend downwards into the gravel layer for 20 to 30 cm or more. Here, as elsewhere, the laterite is ‘soft’ in freshly cut sections, but it rapidly hardens in older exposures. The granule layer is usually a visually distinctive concentration of individual angular quartzite granules ( 1—4 mm) that appear to have been smashed, each supported by the cover sand (*i.e.* matrix supported). The layer is clearly picked out by magnetic susceptibility and luminescence profile data trends. The magnetic susceptibility and luminescence profile trends are considered further within Sect. 3.3 and within the Discussion. However, locally the granule layer can exhibit grain-to-grain contacts, although not closely packed. Above this layer of concentrated granules, the smashed granules become finer, more diffuse, and supported by the cover sand to 0.5 m above the granule layer, above which granules are infrequent throughout a further 1.5 to 3 m thickness of cover sand.

The red cover sand (samples *c*., P18 to P1 in Fig. 7) has frequent scattered, rounded, small quartzite granules which become smaller and less frequent up the section as grain size samples fine upwards and become progressively positively skewed. The luminescence profile data trends suggest that a lower distinct horizon exists (*c*., P18 to P12) with an upper distinct horizon (*c*., P 11 to P1) the top of which is affected by soil development or anthropogenic disturbance (*i.e.* magnetic susceptibility values at P5 to P1). The inversion in quartz OSL data is particularly striking.

#### Khon Kaen (Thailand)

16.50127 to 16.5002° N; 102.7585 to 102.7622^o^ E

**Fig. 8:**
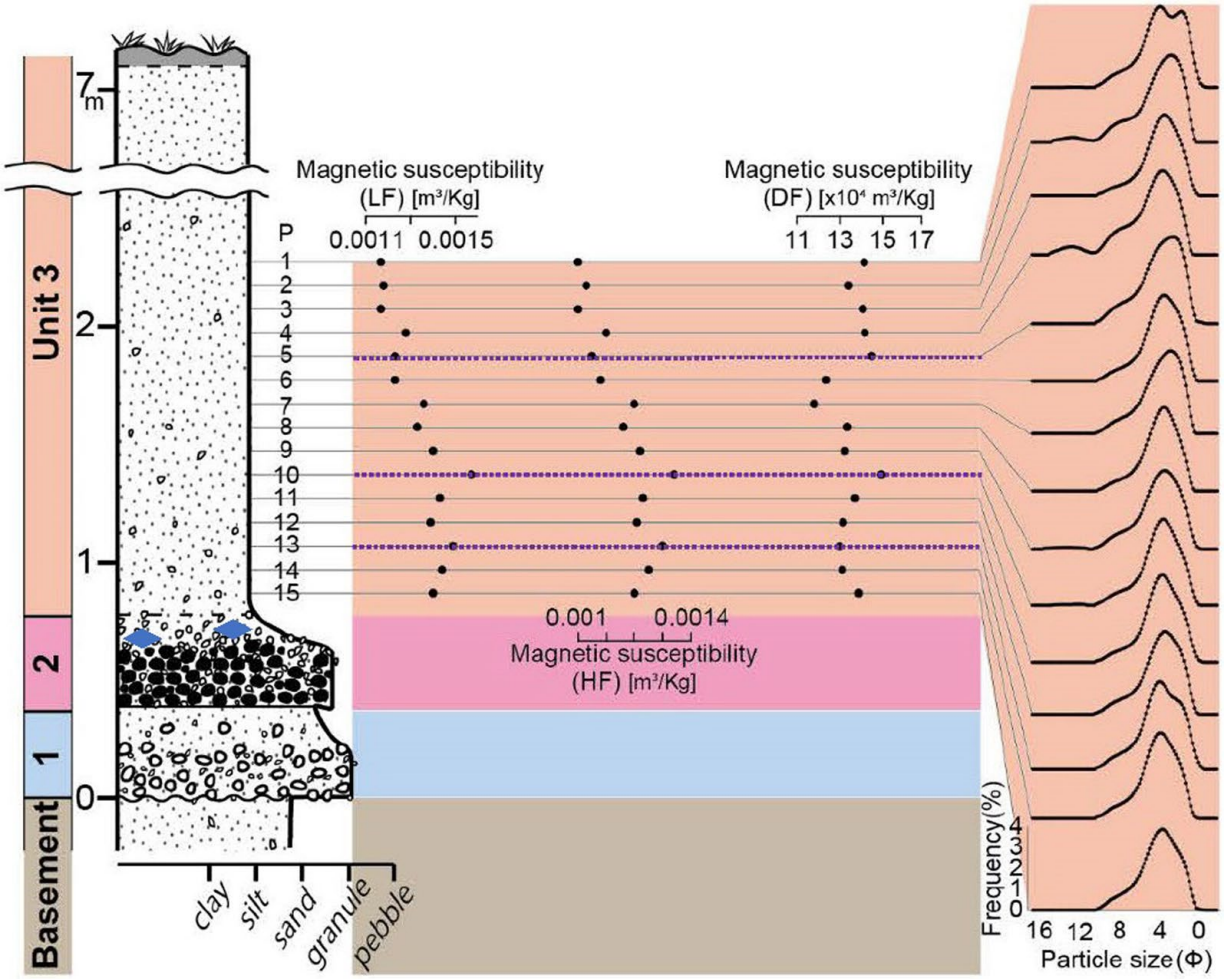
Summary stratigraphy for Khon Kaen. Sample (P) numbers represent the samples mentioned in the text. Mass specific magnetic susceptibility, LF and HF (m^3^/Kg), percentage frequency-dependent susceptibility (DF) and portable OSL and IRSL (count) values highlight the cryptostratigraphy, with grain size curves on the right of the figure. Sub-divisions of Unit 3 are shown as purple pecked lines. Blue diamonds represent tektites

*Basement—weathered local rocks or widespread gravels:* The basement is a reddish purple sandy weathered residual of the lithified rocks below (Cretaceous Maha Sarakham Formation not exposed) that can be weathered to a white clay-rich residuum (Fig. 8). Petrified wood is found in the basement here.

*Unit 1/2: Breccia and gravel:* It is difficult to separate Units 1 and 2 at this location although the lower 1 m thickness of the section is reddish brown, granular and sandy; reminiscent of the (*c*., 20 cm thick) top of Unit 1 at Huai Om. There is no basement fluvial gravel here (unlike Kok Yai) but, above the reddish-brown sand, a lag layer of fine quartz gravel pebbles and granules together with shiny black volcanic (?) pebbles within a sandy matrix appears to be a residuum of the weathered basement, reworked by a geophysical flow, as it also contains angular weathered, friable, sandstone fragments apparently sourced from local basement outcrops. The white quartz pebbles are angular to sub-angular with an admixture of very well-rounded pebbles with a silt matrix. Occasional fragments of petrified wood derived from the basement are also present, as are tektites (Fig. 8), within the laterite, which are common at this location and other sections nearby. This *c*., 1—2 m thick gravel layer is very weakly stratified and lies unconformably along an undular sharp truncation surface cut within the basement. Where thinner, the gravel layer frequently divides into a slightly coarser lower layer (< 0.6 m thick) and a somewhat finer upper layer (< 0.3 m thick) with two gravel stringers in between. The upper layer and the stringers are embedded in reworked sand from the basement containing frequent granules. The two thicker beds largely are unsorted but do show very weak upward fining often with a slight concentration of well-rounded larger pebbles at the base. Bedding is absent or is vaguely present for just a few decimetres along the outcrop, usually picked out by lines of rounded pebbles. The upper contact with the cover sand (Unit 3) is less well defined, often being diffuse. The contact between Units 2 and 3 undulates markedly with wavelengths of several metres, but undulations are not regular. The amplitude of the undulations is small, so the gravel layer does not noticeably thicken and thin as it does at Kok Yai.

*Unit 3: Cover sand*: The cover sand here is similar to that at Kok Yai (Ban Basieo) and up to 6 m thick, but less than 3 m thick at the sampled section. A granule layer in the basal 20 to 50 cm of the cover sand is integral to the cover sand lying above it. The granule layer is laterized in the upper portion of the layer in the undular hollows but not on the high points and laterization can extend down into the top of Unit 2. The granule layer (represented by samples P15 to P13) is a visually distinctive concentration of smashed and sub-angular quartzite granules (1–4 mm) individually supported by the cover sand (*i.e.* matrix supported). However, locally it can exhibit grain-to-grain contacts, although not closely packed. Above this concentrated layer, the smashed granules become finer and more diffuse to 1 m above the granule layer, above which granules are infrequent throughout a further several metres thickness of coarse silt to very-fine sand. A thin laterite (< 100 mm thick) consisting of nodules of sand occurs within the upper 2 m. The magnetic profile data trends suggest that a lower distinct horizon exists (*c*., P13 to P10) with an upper distinct horizon (*c*.*,* P10 to P1) the top of which is affected by soil development or anthropogenic disturbance (P5 to P1).

#### Krahad (Thailand)

15.63075° N; 102.0021° E.

**Fig. 9:**
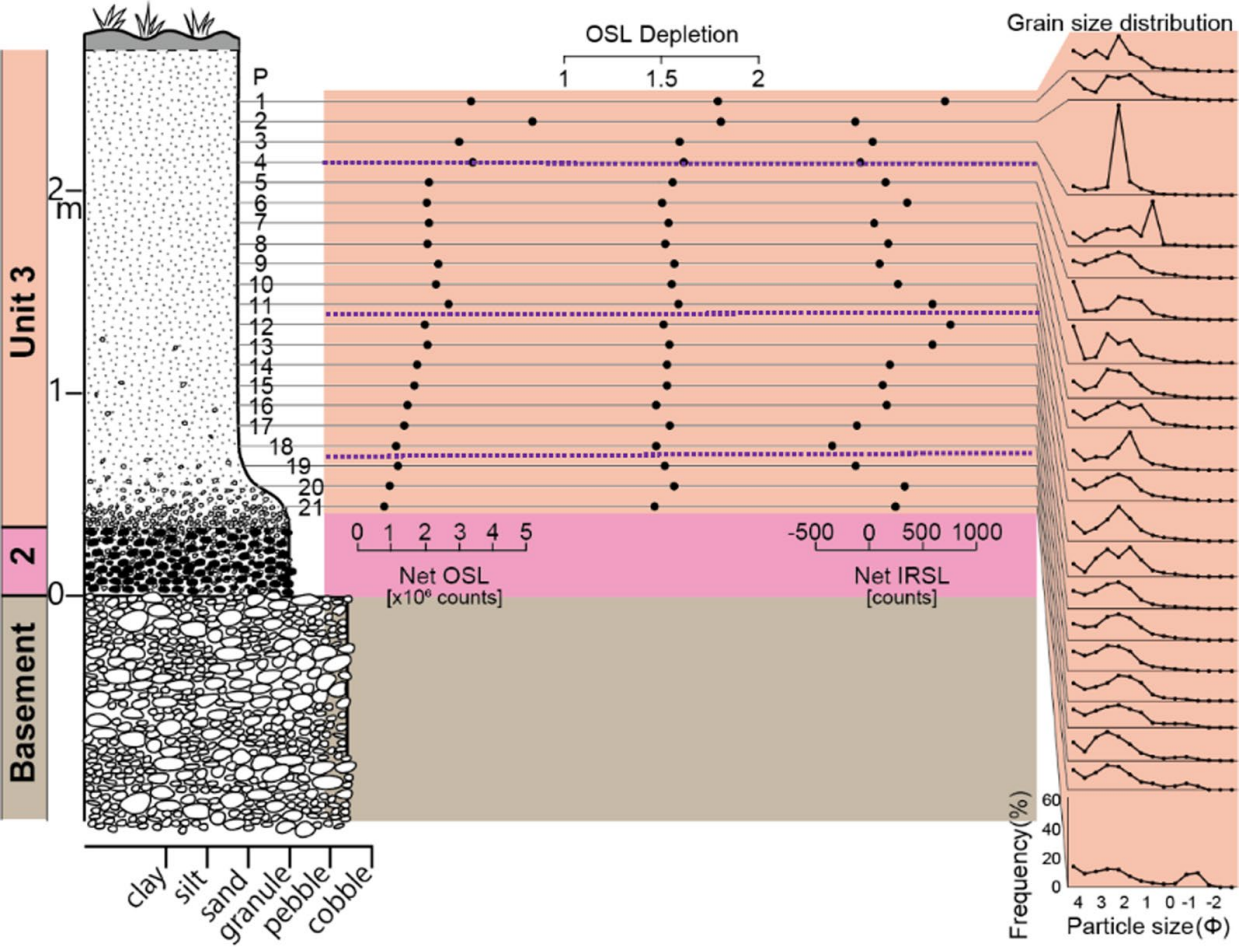
Summary stratigraphy for Krahad. P numbers represents the back dot samples mentioned in the text. Portable OSL and IRSL (counts) values highlight the cryptostratigraphy, with grain size curves on the right of the figure. Sub-divisions of Unit 3 are shown as purple pecked lines

The section here is very similar to that at Kok Yai so full details are not reiterated here. The basement is consistent with the Khok Kruat Formation and Unit 1 (breccia) is absent. An irregular undulating contact, with wavelengths typically of tens of metres, occurs between a large-pebble layer (basement) and a layer of white rounded small-pebbles (Unit 2). Most of Unit 2 at this location is laterized. An undular contact also occurs between the top of Unit 2 and a laterized granule layer above that lies at the base of the cover sand (Unit 3). Significantly, angular to sub-rounded blocks of laterite occur within Unit 2 (below the granule layer of Unit 3), which appear to be rip-up clasts derived by tearing of a prior laterite surface by a geophysical flow. The laterized portion is up to 0.4 m thick within the top of the gravel layer. The granule layer is up to 0.5 m thick (0.30 m for samples P21 to P19 in Fig. 9). The red cover sand (Unit 3) is about 2 m thick (samples *c*., P18 to P1 in Fig. 9). The luminescence profile data trends suggest that a lower distinct horizon exists within Unit 3 (*c*., P18 to P12) with an upper distinct horizon (*c*., P 11 to P1) the top of which is affected by soil development or anthropogenic disturbance (*i.e.* magnetic susceptibility values at P4/5 to P1). Grain size variations at P3 and P4 also indicate disturbance. This division of Unit 3 is similar to that seen at Kok Yai.

### General observations of the gravels (Unit 2) and cover sands (Unit 3)

The above section primarily described the sedimentology and stratification of the deposits which define three units that occur commonly across the region. Although Units 1 and 2 are sometimes thinly stratified, the cover sand (above the basal granule layer) was always visually homogeneous, although smashed granules become finer and more diffuse upwards through the Unit (*e.g.* Huai Om and Khon Kaen sections). Consequently, as well as the magnetic and luminescence measurements, which can reveal cryptostratigraphy, additional measurements of particle density, bulk density and mineralogy were undertaken at several sites to determine if these measures might reveal any changes in the character of the sediments that are not visible to the eye. Particle density determinations obtained down a 11.5 m thick section at Noen Sa-nga, Chaiyaphum, Thailand (15.62762° N; 102.001° E), consisting of the gravel layer and the cover sand, showed no vertical pattern, with a mean value of 2.57 g cm^−3^ (ranging between 2.45 g cm^−3^ and 2.68 g cm^−3^; s.d. = 0.056 for n = 23) consistent with the predominant presence of quartz and quartzite throughout all three Units. Similarly, bulk density profiles through Unit 3 for Wang Pong, Nakhon Ratchasima ($\overline{x }$ = 2.47 g cm^−3^), Mai Chumphon, Yasothon ($\overline{x }$ = 2.62 g cm^−3^) and Khon Kaen ($\overline{x }$ = 2.54 g cm^−3^), all sites in Thailand (see Supplementary Information for locations), show no vertical patterns. XRD results (Kok Yai) for the fines within the gravel layer and cover sand show the presence of quartz, kaolinite, hematite, and plagioclase; no high-pressure minerals were detected. Rather, the oxides are typical of siliciclastic sediments that are highly weathered (high in SiO_2_ (74.32%), Al_2_O_3_ (14.08%), Na_2_O (5.43%), Fe_2_O_3_ (3.62%), and K_2_O in some locations) (Charee et al. 2013). Similarly, inspecting the X-ray and CT-scans of the basal cover sand there is no evidence of lamination at all, rather the base of the Unit 3 sediment appears massive although mottled due to diagenesis (Fig. S1). Rather, the three distinct layers within Unit 3 noted in Figs. 6 – 9 could only be depicted by magnetic and luminescence signatures as is described in the next section.

### Environmental luminescence, grain size, and magnetic susceptibility profiles

In this section, environmental luminescence parameters are used simply to identify changes in the stratigraphy from Unit 1 to Unit 3 that are not visible in the field. Herein, as elsewhere (Bateman et al. 2007), these measurements have been extremely useful in helping to understand the depositional mechanisms, determining the positions of cryptostratigraphic boundaries, and identify mixed or redeposited horizons possibly highlighting mineralogical properties.

Further laboratory analysis including calibration doses are reported in Cresswell et al. (2018b, 2019a, b), which provide additional information for interpreting the portable OSL and IRSL profiles (Figs. 6 - 7 and 9). For the IRSL, all samples show low sensitivity, and hence, the data for apparent dose carry substantial uncertainties. The OSL shows higher sensitivity, with apparent doses consistent with samples where the OSL traps are close to saturation. For the sample at the top of the profile (Fig. 6), the OSL sensitivity is very much higher (biogenic reworking?) than for the samples below it, which explains the higher net OSL counts observed with the portable reader. This difference in sensitivity suggests that these samples are not all from the same depositional unit, despite difficulty in visually defining interfaces within the cover sand units during sampling. The sharp division between sensitivities also suggests minimal mixing between these depositional units, so they are discrete*.*

Considering the full profile at Huai Om, for convenience, sample points are numbered from P1 to P24 top down (Fig. 6), and the luminescence values represent two replicate samples at each point. The reproducibility of the method is reflected in the many coincident replicate points throughout the section. The Net OSL and Net IRSL values are generally high and were used as proxies for variations in depositional processes throughout the stratigraphic column. The interface between the weathered basement and the overlying breccia as defined from visual inspection in the field is shown in Fig. 6. Samples P23 and P24 are within the weathered basement, and the sample P21 was obtained just above the interface. It is evident that the offset of the positions of OSL, IRSL, and magnetic susceptibility signals for samples P21 and P22 represents disturbance: *i.e.* varying degrees of mixing of basement material with the overlying breccia (Unit 1). However, from P20 upwards to P12 both the OSL and IRSL signals represent consistent trends, with OSL values generally lower than the basement values whilst some IRSL values exceed those of the basement. Magnetic susceptibility has a similar behaviour except for an excursion at P14. Visual inspection in the field indicated that this P22 to P12 sequence coarsened upwards from clay-rich to sandier with an ill-defined transition mid-sequence. This supposition is supported by the grain size distributions for Unit 1, which also are distinctively bimodal. The OSL signal indicates that the grain size transition occurs at the ‘kink’ between P17 and P16. P11 to P6B are within Unit 2 wherein both the OSL, IRSL and magnetic susceptibility signals depict distinct trends, offset from the trend of the lower breccia. The samples P6B & A lie across the transition from the granules to the cover sand (Unit 3) above. The difference in the OSL and IRSL values comparing P6A and P6B reflects the difference in measurement technique between the two sample sets, 6A was measured with the samples above as bulk sediment in 50 mm petri dishes, whereas 6B was measured from sieved < 500 µm polymineral grains on 30 mm planchettes which maximized signals from the lower sensitivity samples below and allowed calibration of these samples without further laboratory preparation. The slight variation in OSL and IRSL signals for P6A and for P5 to P2 reflects sampling within a largely homogeneous unit of the cover sand. In contrast, the high quartz sensitivity value for P1 indicates a distinct quartz-rich layer lies above the lower cover sand horizon. P1 in fact represents an upper cover sand horizon that could not be sampled effectively throughout its’ depth due to dense vegetation disrupting the sediment body at elevations above P1. The variability of magnetic susceptibility as deep as P3 may indicate biogenetic disturbance to a depth of *c*., 0.3 m. Other than excursions in the coarser gravel layers, the depletion values (although unusually high) are generally consistent throughout the section, suggesting that variations in clast content and sediment colour were of secondary importance to luminescence sensitivity. The OSL Front values, although higher than the net OSL values, basically mimic the trend of the net OSL trends. Signal ratios (not illustrated) show no evidence of substantive variation in mineral content, a conclusion borne out by SEM determinations which showed a predominance of Si and Al, reflecting the predominant presence of quartz, quartzite, and clay minerals.

In summary, the environmental luminescence, magnetic susceptibility, and grain size profiles define a cryptostratigraphy that is consistent with the field description, but importantly allows sub-division of the cover sand. Specifically, the luminescence assay defined: (i) the position of disturbance at the top of the weathered basement where breccia and basement material (Unit 1) is locally mixed; (ii) the general location of the change from clay-rich sand breccia to the gravel layer (Unit 2); and (iii) the intimate relationship of the granule layer with the cover sand above (Unit 3). The results for samples P6A and P6B indicate probable admixture of the fine cover sand (Unit 3) with the granule bed below. Finally, the upper part of the cover sand is disturbed by bioturbated.

### Undulating interfaces between basement and Unit 1, and between Units 2 and 3

Several authors have commented on the distinctly undulating stratigraphic interfaces within the Quaternary deposits across the region (*e.g.* Moormann et al. 1964; Parry 1992) but without providing explanation, so this issue is explored herein.

At nearly all locations investigated in the present study, where extensive good exposures were present, the interface between the cover sand (Unit 3) and the gravel layer (Unit 2) undulates distinctly over relatively short distances (10’s of metres). Often the interface between Unit 2 and Unit 1 also undulates, but with a lesser amplitude, and undulations can occur between the basement and Unit 1. The undulation can be irregular or frequently an apparent (sinusoidal) regularity is evident with wavelengths of decametres; occasionally significant near-vertical distortion of the interfaces occurs over distances of < 2 m, with any preferred particle orientations also paralleling the distortion. Generally, undulations appear to be flow transverse although some may represent flow-parallel longitudinal troughs. Below, two examples are provided from Thailand and from Cambodia.

**Fig. 10:**
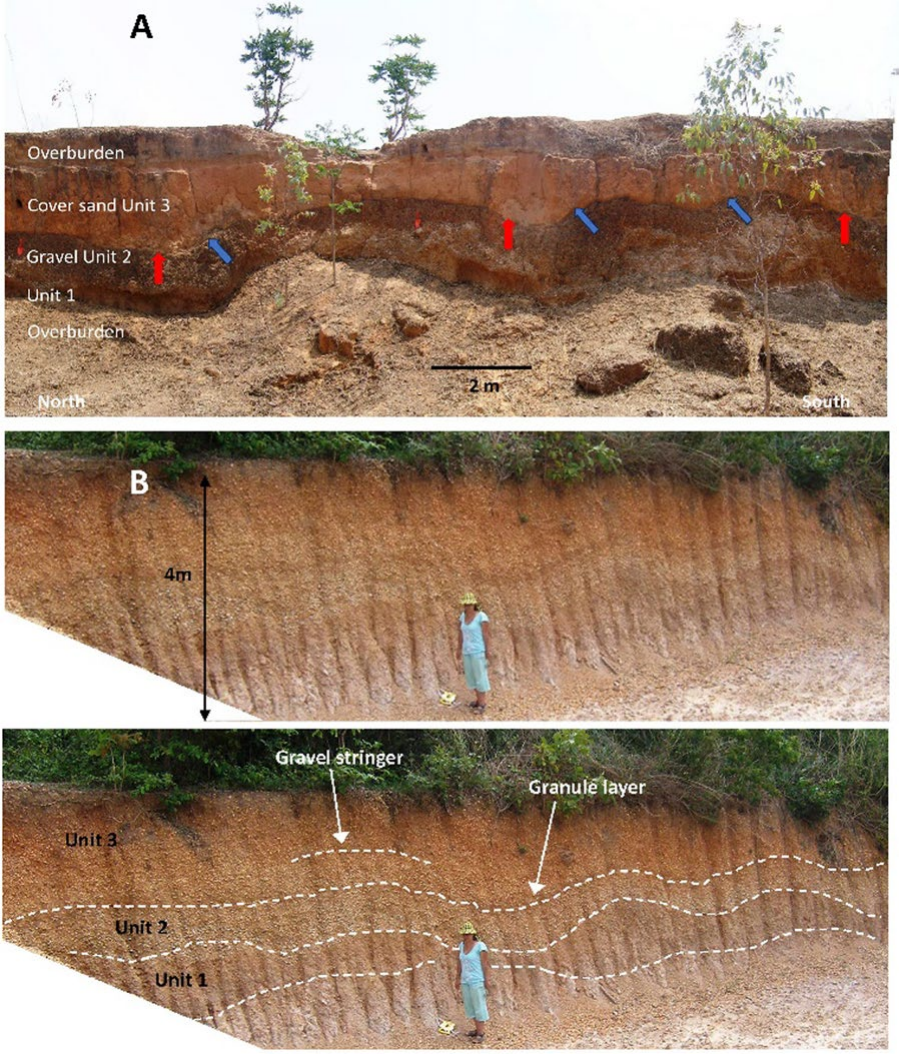
**A** Montage of central section of a 42 m exposure of undulating gravel layer beneath a cover sand. Eroded basement (below red trowels) is lighter colour below the dark-colour gravel layer. The limbs of the top of the gravel layer often are asymmetric with the north-facing limbs being steep (blue arrows), whilst the south-facing limbs usually rise at only a few degrees (red arrows). The predominately unshadowed foreground is overburden. Red trowels are 25 cm long; **B** Section near Stung Treng (13.52204^o^ N; 105.9563.^o^ E) of a 15 m long exposure of light-colour undulating gravel beneath a laterized cover sand. The eroded, weathered, and disturbed basement (Unit 1) is a redder colour, lying above a whitish weathered basement (the latter lying below the lower white pecked line). Unit 2 consists of well-rounded pebble gravel. Unit 3 is a laterized cover sand. The average height and wavelength of the undulations are 0.82 m and 2.82 m, respectively (n = 6)

Near Nakhon Ratchasima, Thailand, Unit 1 consists of reworked basement material and lies on an erosional unconformity above the basement of red and white weathered sandstones. Unit 2 is a distinctive gravel layer. A 250 m long west-facing section (14.85646° N; 102.04071° E) displayed short-wavelength quasi-regular undulations. Regular asymmetrical to symmetrical undulations (Fig. 10A), with heights of 3 to 4 m, occur at the interface between Unit 1, Unit 2 and the cover sand (Unit 3). Unit 2, which is laterized throughout, is thickest in the troughs and thins towards the crests. Feint bedding in Unit 2 tends to parallel the limbs of the undulations.

A 42 m length of this section, orientated approximately 12° east of north, illustrates key points (Fig. 10A). In a south to north transect along this section, the stratigraphy exhibits a distinct undulating interface between Unit 1 and Unit 2, which undulations also occur between Unit 2 and Unit 3 above. The limbs of the top of the gravel layer often are asymmetric with the north-facing limb being exceedingly steep (blue arrows in Fig. 10A), possibly scoured, whilst the south-facing limb usually rises at only a few degrees (red arrows in Fig. 10A). The asymmetry suggests that flow was from the south-eastern quadrant. In section, the apparent bearing of the responsible flow is from south to south-east, but the true bearing could be more easterly.

Near Stung Treng, Cambodia, at a high plateau elevation (Elevation 140 m; 13.52204^o^ N; 105.9563° E) a section cutting through an undulating gravel layer is orientated approximately from 88° to 130° east of north (Fig. 10B). The lower part of the section is a whitish weathered basement sandstone. Based on the lithology of the matrix and the clasts within Unit 1, the unit consists of eroded and redeposited basement. Unit 2 is conformable with Unit 1 and consists of small, well-rounded pebbles, largely unstratified. The base of Unit 3 consists of a pebble-rich granule layer which is conformable with Unit 2 below but fines upwards into a laterized sandy cover sand (Unit 3). Unit 2 thickens slightly in the troughs compared with the thickness of the gravel over the crests of the waveforms (Fig. 10B). The waveform of the bedding plane beneath the gravel layers has a more pronounced amplitude than the corresponding bedding plane above the gravel layers and is sometimes asymmetric with longer stoss-side limbs up flow of the presumed flow direction and steeper shorter limbs down flow. The asymmetry suggests that flow was from the north or north-western quadrant.

### Significance of the association of tektites with Units 2 and 3

**Fig. 11:**
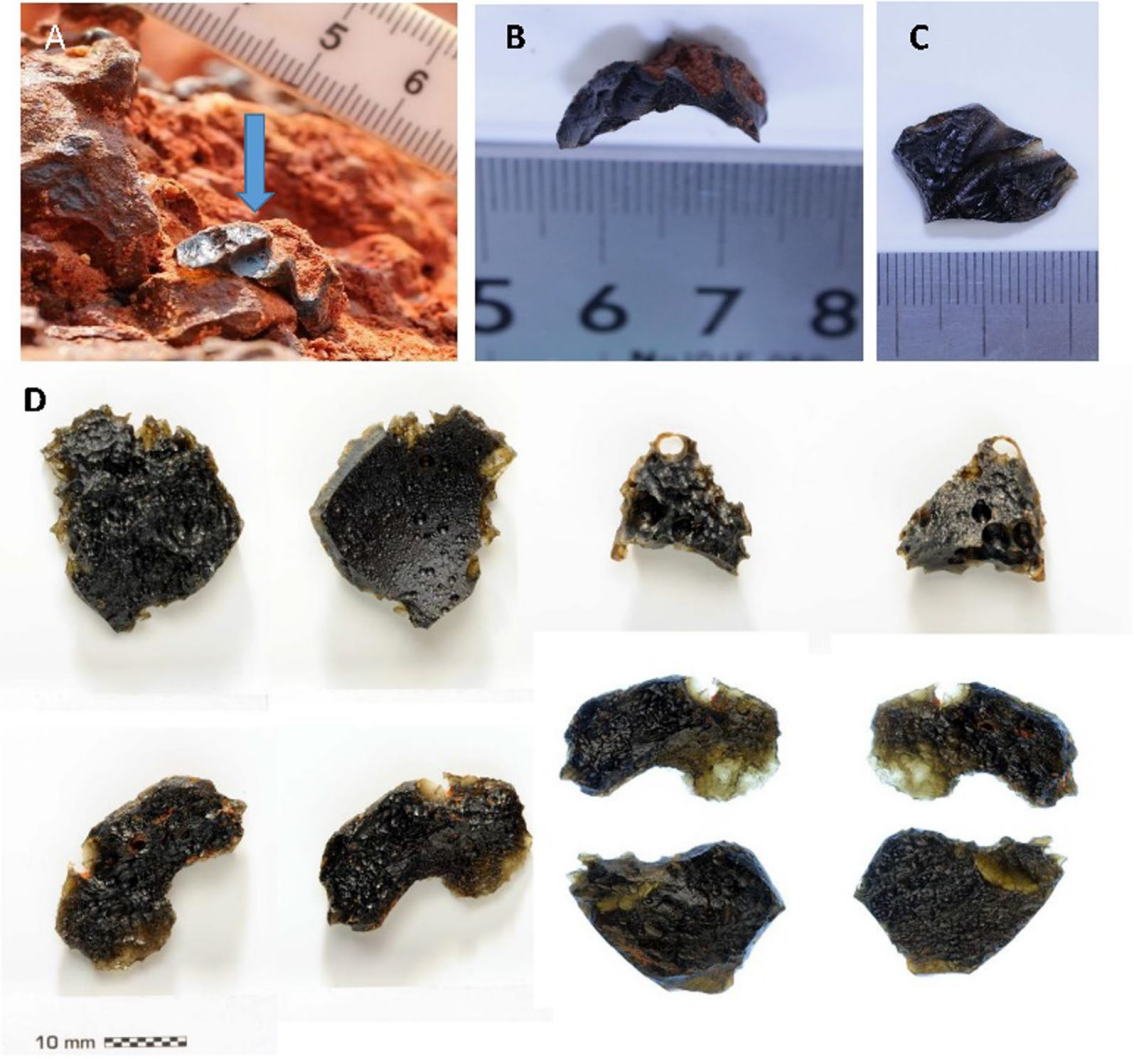
**A** Small tektite (arrowed) in situ, wrapped around and closely capping a small pebble in Unit 2 at Khon Kaen; **B** Tektite in (A) removed from pebble; note concave lower surface that was close-fitting and wrapped around the top of the pebble; **C** Aerodynamic flutings on tektite found at Khon Kaen; **D** Reverse and obverse images (left and right images, respectively) of delicate and partially translucent tektites found in situ in Unit 2 at Zi Mu and Krahad

All sections visited (Table S2) were searched for tektites. Of significance is the fact that all tektites that were recovered in situ were found at the top of Unit 2 or within the upper 200 mm or so of this unit. Very rarely tektites were found within the granule layer at the base of Unit 3 but not at higher locations within the sandy sediments. Tektites were found in Thailand at Kok Yai, Krahad, Khon Kaen, Kalasin, Ban Nong Makhue, and near Haui Om, as well as at other locations. Several tektites were recovered in situ along the top of the gravel layer (Unit 2) at Kok Yai and one at Ban Nong Makhue. At Khon Kaen, tektites were common, around a dozen being found on the top of Unit 2, with two additional examples found, washed down the exposed section and loose on the floor just below exposures. Importantly, one small tektite was found in situ (Fig. 11A), wrapped around and closely fitting a small pebble (Fig. 11B), which shows that the tektite deformed and then solidified during deposition. A further example exhibits aerodynamic flutings (Fig. 11C). A total of six tektites were found at Krahad and one at Kalasin. None of these latter examples were in situ in the section. However, two examples were found embedded in blocks of the slumped lateritic gravel layer (Unit 2) close to the sections. These tektites were shiny, exhibiting primary sharp edges, flutings and vesicles (Fig. 11D top row). Two examples are broken fragments (Fig. 11D top row and bottom right). Importantly, two other tektites are delicate unbroken flakes, transparent at one end (Fig. 11D: bottom left and middle right). These several tektites (Fig. 11) are about 10 mm in size and seem to be fragments of layered tektites. A further tektite was found at the top of Unit 2 at 14.4265° N; 105.2025° E. At the site HO6 that is near Huai Om, several fragments of layered tektites were found in situ from the upper part of the gravel layer (100 mm below the top of Unit 2; Tada et al. 2020). These latter tektites are each several centimetres in size and were concentrated in limited space about 40 cm × 30 cm × 10 cm. This close association implies that these latter examples are fragmented pieces of one large, layered tektite (Tada et al. 2020).

In Vietnam, more than a dozen tektites were collected from the floor of gravel pits at Dong Ha (Table S2) where the Unit 2 is well developed. Within China, tektites occur frequently in the You-Jiang River terrace, Bose Basin, where a red sand overlies a gravel layer. Tektites are also found here in association with archaeological sites (Yuan et al. 1999). The sedimentary sections here have not been studied in detail as part of the current investigations but have similarity to the upper two units noted in Thailand (see following section). At Gaoling Po, tektites are frequent, being scattered on the modern surface, but several were found in situ in the red sand (Unit 3?), 1.2 m above the top of a 1 m thick ferrocrete formed in the top of a gravel layer which may represent Unit 2. At Zi Mu, five tektites were recovered on the surface. At Gan Lian, tektites occur frequently in the cover sands and the gravel layer.

In Laos, tektites are reported by residents as widely distributed and, in this study, tektites were found ex situ at several locations and six were found in situ in Unit 2. Similar to the wrapped pebble in Thailand (Fig. 11A, B), one pebble in Laos had clearly been wrapped around the top of pebbles when molten (not illustrated). In Cambodia, two in situ tektites were found at the top of Unit 2 at Sre Sbov (N: 12.86169° E: 106.194°).

### Optically stimulated dating of the stratigraphy

In 'Introduction', the problems associated with dating the surface Quaternary deposits in the region were noted, especially with regard to application of luminescence dating. Consequently, it is appropriate to consider this matter in the following text (see also Supplementary Information).

Of the 14 sandy samples taken for OSL dating in this study, six (Table S3) were used to define the relative or absolute ages of the upper cover sand of Unit 3, with the remainder (Table S4) used to define the basal granule layer within Unit 3. The unit 3 sands tend to young upwards, but the basal granule layer is not as old as expected, yielding dates ranging from as young as 8.5 ka to 150 ka (Tables S3 & S4). The Tad Huakhon sample (Table S4) is an exception being a sample of the matrix within a 0.7—1.0 m thick pebble bed that lies between the sandstone basement and a basalt flow, and which returned a date of 80 ± 20 ka.

All these results show considerable variability in luminescence sensitivity, dose rates and equivalent doses and dose distributions which can explain the variation in the dates recorded. The site at Hue, Vietnam, showed evidence of mixing between younger material and much older material (see Supplementary Information). For both Huai Om and Hue, the oldest component ages determined from a dose extension analyse are consistent (120 ± 10 ka at Huai Om, 100—125 ka at Hue; Table S4). These ages are considered further in Discussion.

## Discussion

### Origin of the breccia (Unit 1)

The breccia consists of large amounts of local, or regionally derived, lithologies of poorly sorted, angular, rock chips as typified by Unit 1 at Huai Om (Fig. 6) that is weakly stratified. The breccia is interpreted to represent deposits emplaced as a ground-hugging surge. It is evident that the regional impacted rocks dominate the continuous ejecta. Local stripping of regolith together with some erosion of the basement contributes to this unit, although the relative contribution of sources to the breccia has not been explored. The grain size distribution within this unit is often bimodal (Fig. 6).

### Origin of the gravel layer (Unit 2)

The gravel layer appears to have been sorted and thus separated from the lithologies forming Unit 1, as well-rounded quartz pebbles are concentrated within Unit 2, whereas in Unit 1 they are usually sparse or absent. The coarse gravel portion of the unit is unimodal and probably was separated largely by shape from any other local lithological material eroded by the ground-hugging flow, leaving only a tail of finer matrix-filling fractions causing strong negative skew to the grain size distributions. It is also possible that there is a component from the coarser fractions within the ballistic ejecta, not least as tektites occur in the upper 200 mm of this unit. Consisting predominately of well-rounded quartzite whole and fractured pebbles, with few sub-rounded sandstone fragments and occasional laterite rip-up clasts (*e.g*., at Krahad), it is probable that the pebbles were already well-rounded when entrained into the flow. Being hard, the quartzite, although often broken, resists further fragmentation during transport whereas any softer sandstone clasts would have been largely comminuted, so are less common. The basement often contains well-rounded quartzite pebbles or, locally, Early Quaternary river terraces contain rounded quartzite pebbles (Boonchai et al. 2017; Mustoe et al. 2022), which being eroded by the base surge would contribute the pebbles composing the gravel layer. In addition, the modern land surface often exhibits extensive erosional thin lags of quartzite pebbles, so it is likely that the palaeo-land surface also exhibited a pebble lag that once entrained would contribute to the gravel layer. However, the key point to note is that Unit 2 occurs extensively across the region and is thin, wispy, poorly consolidated, exhibiting sedimentology and bedding styles normally associated with catastrophic floods (Carling, 2013) but on a regionally extensive spatial scale.

The general fining upwards and occasional weak layering and preservation of cross-bedding has also been noted in other ejecta blankets (Fisher & Walters 1970; Fisher & Schmincke 1984, Wohletz 1998; Knauth et al. 2005; Osinski et al., 2013). The presence of fragile tektites and ‘wrapped’ tektites within and along the top of Unit 2 is strong evidence for co-deposition of tektites and pebbles as an ejecta. Being so delicate, these flakes would readily be broken by fluvial transport within a gravel bedload, so later mixing of tektites and gravel can be ruled out. All these tektites display clear primary features; there are no abrasion marks or rounding that would indicate fluvial transport. The fact that tektites usually are found within the top 200 mm of Unit 2 can be explained by the fact that: 1) they were deposited towards the end of the depositional event that deposited the quartz pebbles and sandstone fragments. Alternatively, 2) tektites within the ATSF have densities that can be somewhat less than that of quartz and sandstone fragments, leading to differential sorting by density. Chapman et al. (1964), for Australian tektites, reported the typical range in density as 2.44 – 2.43 g cm^−2^ with a minimum value of 2.31 g cm^−2^. Ten determinations of tektite densities for the tektites collected during the present investigation gave a mean value of 2.31 g cm^−2^. So, a degree of density sorting during the deposition of Unit 2 may have concentrated the tektites towards the upper surface of Unit 2. Without further investigation, it is not possible to choose between these two processes of segregation, as particle shape, size distributions, and concentration are also very important in particle sorting in complex geophysical flows, as is noted below.

If Unit 2 consists predominately of a surface-reworked pebble lag, it would be expected that the surface material would be deposited first, before the locally derived basement (Oberbeck 1975), which is clearly not the case given that Unit 2 lies above Unit 1. Alternatively, the ground-hugging frontal shock at the leading edge of the impact blast was responsible for breaking up and depositing the basement-derived breccia layer (Unit 1) before the arrival of the base surge (density current flow) with its entrained pebbles derived from the basement but otherwise segregated from other basement materials (Unit 2). Grain sorting in geophysical flows, including ejecta, whereby coarser grains are segregated above finer grains (Shinbrot et al. 2017; Wright et al. 2020), is a complex process involving grain sorting by density, size, and shape related to strengthen and weakening of sediment transport (Ferguson 2003) including particle bypassing and kinematic sieving effects depending on the concentration of particles in the flow.

### Origin of the cover sand and the granule layer (unit 3)

The cover sand has been considered by some authors to be possibly bioaccumulated, primarily by termites (Löffler & Kubiniok 1991; 1996; Om et al. 2023). However, for reasons of brevity, consideration is given as to why the granule layer at the base of the cover sand cannot be bioaccumulated in Supplementary Information: Termites & Bioturbation. Below, focus is given to the preferred explanation relating the cover sand to the impact event.

The cover sand (Unit 3) at the locations investigated cannot be a weathering product of the basement as the breccia and gravel layer (Units 1 & 2) usually lies beneath it. In turn, the breccia lies above distinctive weathered basement rocks in all cases, as was noted recently in southern China (Wang et al. 2018) and north east Thailand (Tada et al. 2022). The distinct four layers within the cover sand seen in the magnetic susceptibility, grain size, and environment luminescence profiles also indicate that the cover sand is not a simple weathering product of the basement, but the layering represents phases of ejecta plume fall-out deposition and subsequent diagenesis as is explained in section ‘4.6 Depositional Model’. Further, the cover sand is not a loess (silt-size and finer), being far too coarse. Loess contains negligible fine sand and the cover sand contains abundant coarse sand as well as distinctive granules (*c*., 1 mm in size). Classic Chinese plateau loess sequences exhibit no stratification at all, although thin lamination might occur locally (Cilek 2001). Moreover, loess does not generally record size trends in the vertical, whereas the cover sand often is characterized by systematic upward variations in grain size over a couple of metres from the top of Unit 2 throughout Unit 3 with a concomitant increase or decrease in the degree of negative or positive skew in the distributions (Figs. 6, 7, 8 & 9). Importantly, the cover sands at Huai Om contain shocked quartz (Tada et al. 2022).

### Discussion of erosional undulations

Tropical stone lines concentrated by termites are sometimes reported to undulate but over short distances of a metre or so (Young 1976; Williams 2019), but such stone lines occur at shallow depths, a metre or so beneath the surface. The considerable depth of the undulations reported here (several to tens of metres) and the occasional sedimentary structures, including horizontal lamination, isolated clast pods, cross-bedding, and convolutions, within the gravel beds indicate subaerial deposition of the gravel and not biogenic concentration of clasts into a layer beneath a soil cover. Large-scale erosional or depositional undulating topography due to geophysical flows on Earth has been little studied but is generally related to supercritical flows (Carling et al. 2009a, b; Symonds et al. 2016). Specifically, erosional topography due to the surge from volcanic eruptions largely has been reported in the form of longitudinal grooves (Kieffer & Sturtevant 1988; Sparks et al. 1997) although Sparks et al. (1997) reported plunge-pool-like erosional hollows. Crowe and Fisher (1973) and Schmincke et al. (1973) reported possible depositional antidunes in base surge deposits from volcanic eruptions, whilst Brand et al*.* (2016) and Douillet et al. (2013) reported undulating dune-like topography due to deposition by pyroclastic flows, which may be similar to the undulating depositional pyroclastic topography produced experimentally by Breard & Lube (2017). On balance, the undulating interface between the south-east Asian basement and the overlying breccia appears to relate to the base surge stripping the contemporary surface, which is mimicked by undulations in the upper surface of the gravel layers deposited into these scoured hollows. However, caution is advised in applying this interpretation to gravel stringers deposited at shallow depths in those locations where biogenic stone lines are well developed.

In both of the examples presented within Results, the undulating interfaces, defining the bedding planes between the overlying laterized cover sand, the intervening gravel layer, and the underlying breccia (which lies above basement weathered red clay), are reminiscent of an erosional antidune topography developed in the underlying basement with a conformable deposit of gravel from standing waves and subsequent deposition of cover sand above the undulating gravels. Such an interpretation would be consistent with a short-duration geophysical flow first eroding the basement, depositing the gravel and, with rapid cessation of flow, preserving the undulating topography beneath a subsequent drape of sand. Although we are not aware of any studies that produce antidune-like structures in ejecta blankets, Howard (1974) and Oberbeck (1975) reviewed evidence of longitudinal lineations and flow transverse dune-like and hummocky features in meteorite impact ejecta blankets on the Moon. Consideration of bedforms in pyroclastic flows (Self & Wright 1983; Brown & Branney 2004) is informative as the flow structure over sinusoidal topography is physically similar in both air and water (Poggi et al. 2007), as are the bedforms and stratification styles (Moorhouse & White 2016). Antidunes in water flows develop to a maximum height:length ratio (*H:L*) determined by the steepness of the associated standing waves which become unstable and collapse when *H*:*L* > 0.14 (Allen 1984). As antidunes cannot develop steepness greater than the standing waves, *H*:*L* ≤ 0.14 should characterize the steepest antidunes. For the example near Stung Treng (Fig. 10B), twelve estimates of *H*:*L* were made from the geometry of the bedding planes to give an average* H*:*L* value of 0.136 (s.d. 0.028; s.e. 0.008), which indicates that the undulations are consistent with an antidune interpretation.

Conventionally, antidunes are reported as sediment accumulations within water flows (Allen 1984), and, in all cases, they indicate the presence of transcritical or supercritical flow with flow separation occurring near the crests of the steepest bedforms extending over the troughs (Carling 1999). However, such water flows when developed over an initially plane, compact, erodible surface cause quasi-sinusoidal erosion of the bed leading to a wavy bed level (Ali & Dey 2016). In such cases, the bedform topography is fully erosional or erosional with a depositional component on top (Carling et al. 2009a, b). As the ratio of amplitude to wavelength is increased, the positional of the reattachment point of the separated flow above the bed shifts upstream defining a short recirculation zone (Ali & Dey 2016), in which bed erosion is at first enhanced, but on the falling stage this location is one of preferred deposition on the downstream flank of the bedform, as is the case with the example illustrated in Fig. 10A. In other locations, the thickening of the basal gravel bed in the troughs of undulations or the accumulation of broken blocks of laterite in scour hollows (not illustrated) also indicates strong unsteady geophysical flow processes acting to erode a laterized surface regolith as well as the weathered basement.

The above analysis necessarily is based primarily on the understanding of the development of undular beds beneath water flows, and the interpretations related to high-velocity airflow should be viewed with caution (Douillet 2021). The development of unsteady flow, such as standing waves, within strong gaseous flows has been modelled for volcanic eruptions (e.g. Orescanin et al. 2010), but the implications for meteorite impact studies have yet to be determined. Nonetheless, taken together, this stratigraphic evidence points to impact-induced base surge-induced bed scour and erosional undulations beneath air flow, but water was not involved. In principle, the direction of the flow might be inferred from the asymmetry of the undulations. Where asymmetry is unclear, the main direction of flow should conform with the true dip of the undulations. However, this issue was not addressed in the present study as the largely two-dimensional sections revealed an apparent dip, which may differ significantly from the true dip.

### Discussion of luminescence dating

The apparent paradox of fragile and ‘wrapped’ tektites deposited within sediments of an apparent young age remains unresolved. The OSL ages (Table S3 & S4) in the granule layer are only a fraction of the estimated mean residence time, consistent with moderate to strong downward mixing (Heimsath et al. 2002; Furbish et al. 2018a; Williams 2019) of the overlying cover sands at some locations into the interstices of the granule layer. The OSL ages obtained in this study are significantly younger than the 700–800 ka age of the meteorite impact resulting in the tektite-rich gravel layer. The occurrence of ‘overly young’ ages can be explained, in part, by the analysis of samples from Hue which indicated that the traps responsible for the dose extension measurements may not be thermally stable at environmental temperatures in the region (see Supplementary Information), and thus may produce equivalent doses that result in reduced ages less than the physical ages of these sediments. Similarly, Wang et al., (2018) argued the age of basal deposits (his Units T1 and T2 therein) is older than conventional OSL dating can indicate. Significantly underestimates of the age of early Quaternary events dated using TT-OSL would be expected at elevated environmental temperatures in the region of 25°—35° C and above (Supplementary Information), so additional study is warranted. So far, further investigations into trap stability and age extension of the current samples (Tables S3 & S4) have yet to resolve the paradox (Cresswell et al. 2022) although investigations elsewhere have pushed the age determination of TT-OSL back to 715 ka (Faershtein et al. 2020) showing promise for future dating of the meteorite impact event using luminescence dating.

Previous investigators also have noted that the stratigraphic ages of many Australian tektites (K–Ar dated to 750–800 ka), recovered from Australia, are around 7–24 ka (Lovering et al. 1972; Chalmers et al. 1976, 1979; Glass 1978), so the young stratigraphic ages (Tables S3 & 4) are not unusual. Koeberl (1992) noted that Muong Nong-type tektites are sometimes deeply eroded, mainly by interaction with water; thus, the paradox of tektites with ages of 750–800 ka within much younger sediments could be explained by erosion and re-deposition of the tektites from their original settings within more recent strata. However, such a conclusion in the present context would be at odds with the preservation of ‘wrapped’ and fragile tektites that could not have withstood fluvial transport.

The presence of fragile and ‘wrapped’ tektites within the gravel layer and the intimate association of the granule layer and cover sand with the underlying gravel layer indicate that these units are relatively old (*c*., 800 ka BP as determined from tektite geochronology) despite the apparently young OSL ages indicated just above. Although it is possible that the Khon Kaen and other ‘wrapped’ tektites could have been transported into place with their associated pebbles a long time after the impact event, it is considered unlikely that the tektites would have remained in contact with the pebbles if transported far. Similarly, it was concluded that the fragile translucent tektites must have been deposited contemporaneous and intimately with a rapidly depositing flow of gravel; otherwise, their fragile nature would not have survived reworking. Thus, the deposition of the gravel layer (Unit 2) (associated with the fragile tektites) and the tektites themselves can be considered coaeval, and so of the same age*.* This latter conclusion is important, as the presence of tektites previously has not been considered sufficient to identify the ejecta layer (Fiske et al. 1996; Koeberl & Glass 2000).

In summary, the Holocene dates recorded for this investigation, as well as by prior studies, are inconsistent with the presence of tektites co-deposited with other lithologies in the deposits. Further, detailed high-resolution measurements of the total luminescence intensities throughout the vertical profile of the granule layer and the upper two cover sand units using portable OSL readers will be necessary to reveal any mixing structure, as well as intensity (Furbish et al. 2018b). In addition, the temporal exponential decrease of ^26^Al/^10^Be ratios in the profile of the cover sands could be examined to determine the burial histories, as this approach has proved successful in an examination of burial histories of Quaternary sediments in South Korea back to the Brunhes–Matuyama reversal (Lebatard et al. 2018).

In the same area where tektites are common in southern China (Guangdong and Guangxi provinces), Wang et al. (2018) briefly report a reticulate mottled red ‘earth’ (grading up into a more uniform-coloured red ‘earth’) deposited unconformably above the weathered basement. Wang et al. (2018) note that this stratigraphic unit is characteristic of the region, but it is absent further north where tektites are not reported. The ‘earth’ is capped by a gravel bed (very similar to that reported in this study) which in turn is overlain by a cover sand. In terms of the stratigraphic position, the ‘earth’ is coincident with the breccia noted at Huai Om (and elsewhere), which is mottled in the lower part of Unit 1 and exhibits a more even in hue in the upper part (Fig. 7). Importantly, Wang et al. (2018) place the base of the red ‘earth’ at 730 ka, whilst the cover sands yielded OSL dates very similar to those obtained in the present investigation (see below). One basal cover sand showed signal saturation, and so was undatable, whilst a shallow sample produced an age of 21.35 ± 0.73 ka. Otherwise, the De (equivalent dose) values and OSL ages reported by Wang et al. (2018) increase with increasing depth, with three dates just above the top of gravel beds yielding dates of 79.25 ± 1.65 ka, 116.42 ± 2.86 ka and 132.73 ± 10.36 ka.

From other studies, including electron spin resonance, the reticulate red ‘earth’ is formed from 730 to 400 ka, and the uniform red earth developed from 400 to around 100 ka (Guan et al*.*, 1993; Yang et al. 1996; Yu et al*.*, 1996; Xia & Yang 1997; Sui & Yao*.*, 2000; Lai et al. 2005). The interface of the mottled red ‘earth’ and the weathered basement is placed using electron spin resonance as early Pleistocene (1230 ka) by some authorities (Jiang et al. 1997; Yin & Guo 2006). However, in some locations a gravel layer is reported between the basement and the red ‘earth’ (Li & Gu 1997) which has been assigned to the Middle Pleistocene (Shi et al. 1989; Yang et al. 1996; Gu, 1996; Sui & Yao 2000; Yang et al. 2005). The dates reported above tend to young upwards, nevertheless, there are clear inconsistencies in published dates for the older sediment units above the interface with the weathered basement. Thus, given the uncertainty in dating, such as luminescence signal saturation, the red ‘earths’ in southern China may represent deposition at a time commensurate with the timing of the deposition of the breccia, gravel layer, and cover sands in Thailand and Vietnam. Further investigation is clearly warranted.

### Depositional model

Although there is local variability in the sedimentology, the following descriptions of the strata reasonably apply to the ejecta sequence locations noted within Fig. 3A. The approximate area of the ejecta blanket explored to date is at least 300,000 km^2^ and may have extended further  into southern China, for example (Fig. 3B).

**Fig. 12:**
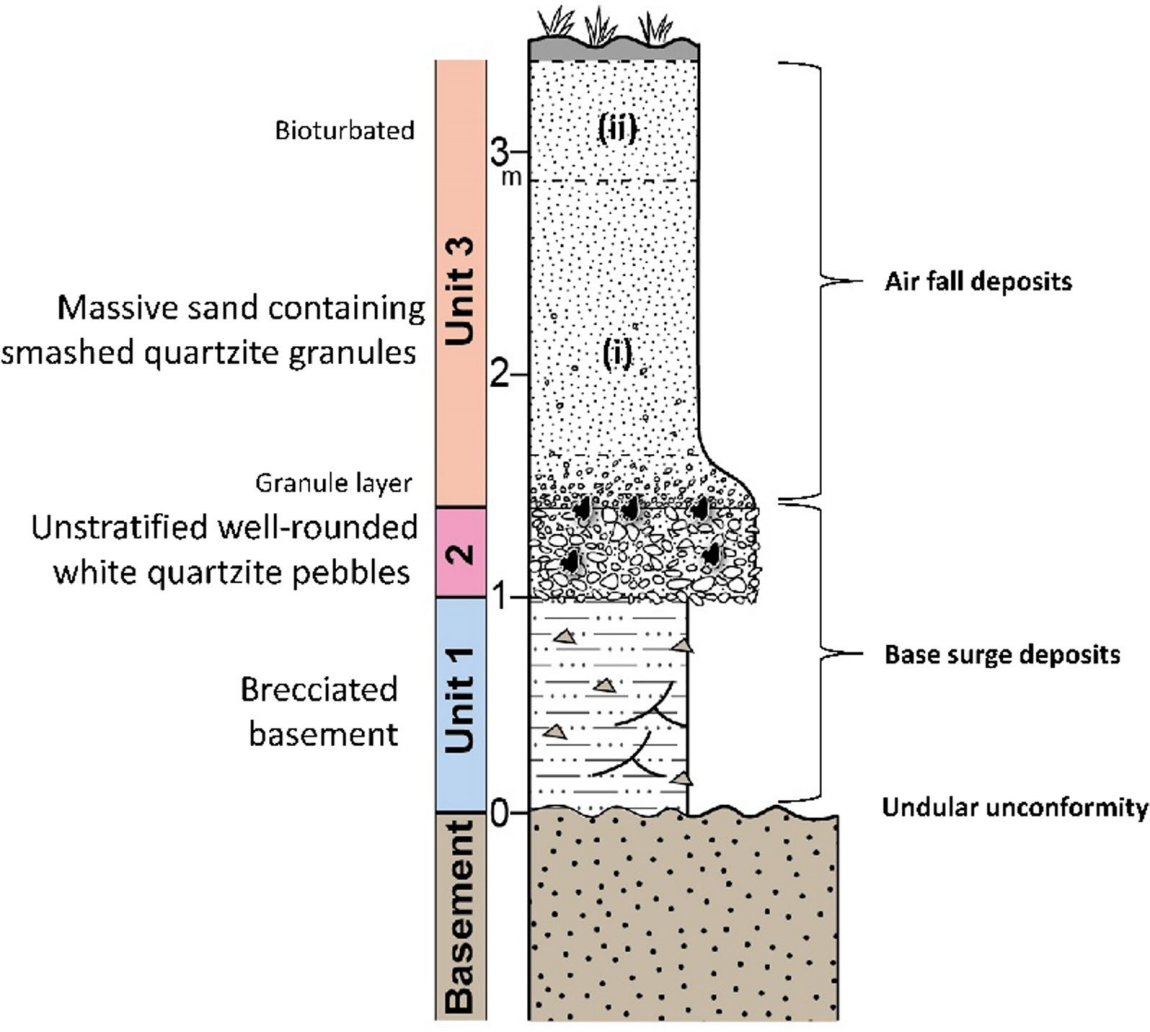
Summary depositional sequence of meteorite impact deposits in Mainland South-East Asia. The basement is undefined as it can vary across the region. The unconformity with Unit 1 is often distinctly undular (as can be the interface between Units 2 and 3). The thicknesses of Units 1 and 2 as shown are typical, although Unit 3 can be several metres thick. Unit 1 is commonly laminated fine to coarse angular sand beds containing bedrock chips, local cross-bedding, and the bedding grain size varies non-systematically upwards. Unit 2 consists of unstratified to weakly stratified, well-rounded white quartzite pebbles with frequent (black) tektites. Unit 3 is unstratified (i.e. massive), but the base consists of a distinctive dispersion of smashed quartzite granules that are matrix-supported (the same fine to coarse sands as occur above); the grain size of the unit sands varies only slightly upwards, although scattered smashed granules become rarer and smaller up-section. The top may be bioturbated

The characteristic sedimentary sequence related to the meteorite impact is as follows (Fig. 12):

- Interface with weathered basement; b) breccia (Unit 1); c) gravel layer (Unit 2); d) granule layer; e) cover sand(s) (Unit 3) (*i.e.*, Unit 3 consists of the granule layer and the cover sand).
- Basement interface: The undulating interface (metre to 10’s of metres wavelength) between the weathered basement and the overlying breccia is often characterised by short scour hollows (1 m—10 m long and up to 2 m deep). The large-scale undulation is interpreted as an initial lateral blast basal surge scoured surface, locally producing regular undulations akin to erosional antidunes. The small scour hollows, often filled with broken laterite, represent local changes in the intensity of the erosional process.
- Breccia (Unit 1): This unit is sometimes absent or indistinct (Fig. 4). However, where present it is termed a breccia because it is largely constituted of reworked weathered basement fragments that can be mostly unstructured in the lower part (although thinly laminated higher in the section), as is typified at Huai Om (Fig. 6). It is occasionally cross-bedded and contains rip-up chips of basement. This material is probably related to deposition from the lateral wind of vapour expansion (Quintana et al. 2018; Schultz and Quintana 2017), following immediately after surface scouring by the initial blast as proposed for Unit 1 at Huai Om by Tada et al. (2022).
- The gravel layer (Unit 2): Although sometimes this unit can be dominated by angular fragments of basalts or laterite, here this unit is termed a gravel layer to distinguish it from Unit 1. It characteristically contains white well-rounded whole and smashed quartzite and angular sandstone pebbles that originally could have constituted a surface lag layer of concentrated weathering-resistant clasts derived from the basement sandstones (not least because the weathered basement does contain well-rounded quartzite pebbles; (Racey et al. 1996; *e.g.* 15.2958° N; 105.4738° E; Thailand; 13.17638° N; 106.17243° E; Cambodia). The distinct pod-like infillings of scour hollows by gravel, the rapid thickening and thinning of the gravel layer, the presence of local sorting, especially cross-bedding (*e.g*., Fig. 6), indicate that immediately following the impact, a near-surface gravel lag was transported and deposited by a ground-hugging geophysical flow, as noted in other layered ejecta deposits where cross-bedding has been observed (Fisher & Walters 1970; Fisher & Schmincke 1984; Melosh 2004; Osinski et al., 2013). The abundance of tektites in this layer (usually close to the upper surface) some of which are fragile and ‘wrapped’ (and so cannot be reworked) demonstrates contemporaneous deposition of the gravel with the tektites, most likely as the gravel layer stabilized. However, deposition was rapid as usually there are no distinct pebble orientations and often no clear fining-up fabric. A high-energy environment is indicated by the poor sorting, lack of stratification within the gravel bed, and the many smashed (consequently angular pebbles) that would otherwise be rounded, and so the gravel layer is regarded as fluidized ejecta. This layer is the latter phase of the base surge wherein sorting, as described above, has separated the coarser, rounded, quartzite gravels. However, some high-angle ballistic ejecta, sourced from the basement melt, is incorporated in the form of tektites.
- Granule layer (base of Unit 3): This dispersion of smashed granule-size quartzite clasts in a sandy matrix is interpreted as the coarsest air-fall ejecta deposited in the final phase of deposition from the high-lofted ejecta curtain. The sandy matrix consists of the finer air-fall deposits. This interpretation is consistent with the environmental luminescence profiles, for example, from Huai Om (Fig. 6) and, as such, this layer properly is an integral part of the cover sand and so is considered part of Unit 3.
- Cover sand (Unit 3): The granule layer rarely exhibits a sharp contact with the cover sand above, but more usually the contact is gradational with quartzite granules becoming rarer and smaller higher in the cover sand profile. The cover sand exhibits four cryptostratigraphic layers defined by mineral magnetic and luminescence signatures. The granule layer represents the coarsest air-fall ejecta, followed by sandy air-fall ejecta deposit which has sparse fine granules, above which is a finer air-fall ejecta with fewer smaller granules. Taken together these deposits constitute horizon (i) (Fig. 12), fining upwards from granules. The uppermost horizon (ii) (Fig. 12), is bioturbated and may otherwise be the same deposit as horizon (i), but frequently this uppermost horizon is devoid of fine granules. Taken together, these horizons are related to the final phase of fall-out of fine ejecta and thus represent the end of the impact-related deposition.

The deposits ((d) and (e) above) are similar, indeed analogous to the massive, structureless fine deposits that are also recorded as due to air-fall from collapsing volcanic pyroclastic columns (Dellino et al. 2019; their Fig. 11) being deposited above, or in close proximity to, poorly bedded, coarse, traction deposits ((b) above); the latter due to ground-hugging density flows. These units, in close vertical association, reflect the contemporary nature of the two depositional styles after explosive eruptions specifically (Gueugneau et al. 2017) and meteorite impacts more generally (Oberbeck 1975).

## Conclusions

The primary objective was to describe the ejecta layers associated with the ATSF across mainland South-East Asia whilst the aim of the study was to understand the nature of the sedimentary signature associated with the meteorite impact event. Within that context, the surface Quaternary sediments covering large areas of mainland South-East Asia represent the ejecta from a large meteorite impact. Segregation during transport by the basal surge led to deposition of a breccia layer consisting of reworked local basement rocks (Unit 1), followed by deposition of a distinctive gravel bed (pebbles) (Unit 2) which contains shocked quartz. The unsteady nature of the surge resulted in distinctive undulating eroded interfaces between the basement and the ejecta units. The gravel bed frequently contains tektites that were *co-deposited* with the pebbles, mainly at the end of the surge such that tektites, associated with ballistic ejecta, occur most frequently at the top of the gravel bed. Fallout of ejecta from a high-lofted plume followed, consisting initially of the coarser lofted granule-sized fractions, but this deposit rapidly fines upwards to become a relatively homogeneous silt to coarse sand bed (Unit 3) up to several metres thick, the top 1 m or so of which is bioturbated. Thus, the initial hypothesis 'Surface Quaternary sediments across a wide area of mainland South-East Asia represent the effects of a regionally significant meteorite impact' is confirmed. The findings of the study may assist in identifying ATSF ejecta layers further afield, and similar depositional models may assist in identifying other impact sites worldwide.

As well as the distinctive widespread distribution of impact-related Units 1, 2, and 3, the incorporation of both fragile and ‘wrapped’ tektites within the gravel of Unit 2 is a key stratigraphic indicator of the co-deposition of tektites and the gravel. Consequently, the formation of the gravel layer can be related directly to the impact event such that most tektites were not incorporated into the gravel at a later date.

OSL dates for the base of the ejecta plume (Unit 3) are late Quaternary and which, given that Unit 3 is interpreted to be deposited immediately following after Unit 2, seems incompatible with the expected age of the tektite-bearing gravel layer (*c*., 800 ka) immediately below. However, it is possible that bioturbation has caused mixing to occur in the vertical at some locations between older sediment and younger material. Of greater significance, at the elevated environmental temperatures experienced by these sediments the thermal stability of both quartz dating signals and signals from deeper traps associated with TT-OSL and other quartz age extension signals may be insufficient to identify early Quaternary ages. To overcome this limitation, it would be useful to examine similar deposits from a range of different environmental settings and to conduct further kinetic studies of the thermal mean lives of the available dating signals under different regimes.

## Supplementary Information

Supplementary material 1.

## Data Availability

A Supplementary Information file accompanies this manuscript. Reasonable requests for the provision of data can be directed to the first author.
