# Supplementary material for: Regionally extensive ejecta layer of the Australasian tektite strewn field: the MIS 20/19 large meteorite impact in mainland South-East Asia
Source: Prog Earth Planet Sci. 2024 Nov 20;11(1):58. doi: 10.1186/s40645-024-00660-9 (PMC11576802; doi:10.1186/s40645-024-00660-9)
Supplement: Supplementary file 1 — Supplementary material 1. [file 40645_2024_660_MOESM1_ESM.docx]

**Supplementary Information for: Carling et al. Regionally extensive ejecta layer of the Australasian tektite strewn field: the MIS 20 large meteorite impact in Indochina.**

**Supplementary Method: Micro-XRF analysis**

An Eagle III micro-XRF system (EDAX Inc, USA) based at the National Oceanography Centre, Southampton, U.K. was used to analyze all samples. This bench-top system has a large sample chamber that can operate either at atmospheric pressure or in vacuum. The instrument is fitted with a rhodium micro-focus X-ray tube that can run up to 50kV and 1mA and is coupled to a 40 μm polycapillary optic that transmits a high intensity X-ray beam. The proprietary Varifocus™ option allows four possible irradiation spot sizes at the sample surface of 40, 100, 197 or 277μm.  X-ray detection is via a 30 mm^2^Si (Li) detector that has an energy resolution of 139 eV at MnKα. The X-ray system is downward looking and includes two colour CCD video imaging cameras (10X and 100X) and a high precision (±10 μm) motorized XYZ stage that allows non-destructive, simultaneous analysis of elements from Na through U.  All analyses were based on 100 second counts.

*Supplementary Table S1: Summary element composition of the cover-sand (Unit 3) within five samples. Data source is Sayngam et al****.*** *(2013).*

| Element | Na_2_O | MgO | Al_2_O_3_ | SiO_2_ | SO_3_ | K_2_O | CaO | TiO_2_ | MnO_2_ | P_2_O_5_ | Fe_2_O_3_ | NiO | CuO | ZnO |
| --- | --- | --- | --- | --- | --- | --- | --- | --- | --- | --- | --- | --- | --- | --- |
| YT1 | 0.017 | 0.004 | 11.444 | 83.763 | 0.116 | 0.433 | 0.060 | 0.563 | 0.012 | - | - | - | - | - |
| YT2 | 0.022 | 0.010 | 5.777 | 87.520 | 0.079 | 0.553 | 0.131 | 0.846 | 0.014 | 0.214 | 4.845 | 0.002 | 0.003 | 0.001 |
| YT3 | 0.017 | 0.830 | 18.942 | 75.355 | 0.112 | 0.358 | 0.081 | 0.455 | - | 1.033 | 2.814 | - | 0.001 | 0.001 |
| YT4 | - | 0.895 | 14.881 | 77.491 | 0.272 | 0.503 | 0.083 | 0.686 | 0.010 | - | 5.180 | - | - | - |
| YT5 | 5.921 | 0.959 | 10.442 | 76.824 | 0.118 | 0.325 | 0.025 | 0.585 | 0.006 | - | 4.795 | - | - | - |

Quantification of the data is affected using the Eagle Vision™ software (EDAX) that incorporates a fundamental parameters (FP) option as well as the possibility of including standards or reference samples. The FP quantification approach uses fundamental atomic parameters and modelling of X-ray excitation, sample interaction, and detection processes to quantify sample composition. The advantage of FP quantification is that it drastically reduces the number of standards required for analysis and allows for the influence of X-ray focusing optics on the exciting spectrum to improve the accuracy of quantification results for micro-XRF analyses. Reference samples have been incorporated into the calibration to improve the analytical response.

**Field site sedimentary descriptions**

Notes on the sedimentology and stratigraphy were made 142 sedimentary exposures the locations of which are provided in Table S2. Stratigraphic logs were made at around 50 of these locations, which data are not included within this Supplementary file.

*Table S2: Locations visited during this study.*

| No^1^ | Location | Northing^2^ | Easting^2^ | Altitude^3^ (m) |
| --- | --- | --- | --- | --- |
| 1 | Ban Dongbang | 15.27197 | 102.8041 | 179 |
| 2 | ? | 15.28422 | 102.8657 | 188 |
| 3 | Chok Dee | 15.37788 | 102.7683 | 128 |
| 4 | ? | 16.54541 | 102.671 | 216 |
| 5 | near Kalasin | 16.49808 | 103.5126 | 164 |
| 6 | near Chi River | 15.98171 | 104.0452 | 120 |
| 7 | near Burinam | 16.03404 | 103.9078 | 158 |
| 8 | Burinam | 14.92 | 103.1261 | 157 |
| 9 | near Burinam | 14.96107 | 103.1193 | 147 |
| 10 | near Khorat town | 14.35623 | 102.0405 | 257 |
| 11 | Wat Pha Sai Chom Sa Ma Ta | 16.12578 | 102.5999 | 229 |
| 12 | Stung Treng gravel pit | 13.51854 | 105.9493 | 74 |
| 13 | Gaolingpo | 23.56712 | 107.1992 | 146 |
| 14 | near Gaolingpo | 23.56778 | 107.2025 | 128 |
| 15 | Zi Mu | 23.84871 | 106.6456 | 156 |
| 16 | Yang Wu | 23.85345 | 106.6437 | 156 |
| 17 | Da Hua Chang | 23.85343 | 106.6666 | 189 |
| 18 | above Nan Po Shan | 23.85023 | 106.6684 | 208 |
| 19 | brick yard | 23.35225 | 106.6661 | 162 |
| 20 | Gan Lian | 23.63602 | 107.0848 | 117 |
| 21 | Ou Kreng | 13.00495 | 106.1353 | 53 |
| 22 | Opongmoan section X | 13.40166 | 106.2417 | 70 |
| 23 | intersection to Ratanakiri | 13.43181 | 106.0717 | 121 |
| 24 | Opongmoan section Y | 13.41609 | 106.2581 | 131 |
| 25 | Opongmoan section Z | 13.40717 | 106.2442 | 159 |
| 26 | Opongmoan section A | 13.4017 | 106.2279 | 149 |
| 27 | Phumi Thalabarivat | 13.5562 | 105.9347 | 48 |
| 28 | Mekong terrace | 13.6289 | 105.9156 | 69 |
| 29 | Mekong A | 13.68702 | 105.8847 | 69 |
| 30 | Mekong B | 13.69239 | 105.9657 | 77 |
| 31 | Mekong C | 13.75198 | 105.8664 | 102 |
| 32 | Mekong D | 13.92729 | 105.946 | 72 |
| 33 | Krala Preah | 13.83605 | 105.908 | 76 |
| 34 | Mekong E | 13.81792 | 105.8759 | 76 |
| 35 | Mekong F | 13.58353 | 105.9334 | 55 |
| 36 | Stung Treng new bridge | 13.52178 | 105.9436 | 80 |
| 37 | south of Kratie | 12.23815 | 105.9658 | 39 |
| 38 | south of Kratie | 12.23979 | 105.9624 | 42 |
| 39 | south of Kratie F | 11.88054 | 105.7694 | 23 |
| 40 | Ban Ba Seo | 15.62354 | 101.9985 | 208 |
| 41 | Ban Nong Ya Khaonok | 15.61115 | 101.9088 | 228 |
| 42 | Chi drainage | 15.6112 | 101.9271 | 218 |
| 43 | Mun drainage | 15.22418 | 101.6283 | 257 |
| 44 | Mun sand pits | 15.03568 | 102.2923 | 156 |
| 45 | Mun sand pits NR1 | 14.9971 | 102.2463 | 169 |
| 46 | Mun sand pits | 14.85075 | 102.117 | 239 |
| 47 | near Fossil Wood Museum | 14.85891 | 102.0311 | 279 |
| 48 | SSK3 C | 13.80444 | 102.2314 | 61 |
| 49 | HO3 | 14.52194 | 105.0058 | 212 |
| 50 | HO6 | 14.4265 | 105.2025 | 171 |
| 51 | HO7 | 14.6972 | 105.3769 | 166 |
| 52 | K4 Khon Kaen | 16.50127 | 102.7585 | 211 |
| 53 | K3 Khon Kaen | 16.5002 | 102.7622 | 218 |
| 54 | Y2 Yasothon | 15.9823 | 104.0379 | 129 |
| 55 | HO Huai Om | 14.57915 | 105.2754 | 188 |
| 57 | DH9-1 | 16.69389 | 106.7048 | 516 |
| 58 | DH2-2 | 16.92695 | 107.0421 | 28 |
| 59 | DH3 | 16.92514 | 107.0357 | 28 |
| 60 | DH7 | 16.82548 | 106.9423 | 31 |
| 61 | DH1 | 16.78533 | 107.103 | 17 |
| 62 | DH1.5 | 16.79121 | 107.1065 | 23 |
| 63 | H10 | 16.48833 | 107.4739 | 13 |
| 64 | H0 | 16.41386 | 107.547 | 14 |
| 65 | KT-3 | 14.93668 | 107.7174 | 661 |
| 66 | KT-4 | 14.62382 | 107.828 | 32 |
| 67 | KT-7 | 14.86817 | 107.698 | 638 |
| 68 | SK3a | 13.82014 | 102.2326 | 57 |
| 69 | SK3c | 13.81661 | 102.2313 | 52 |
| 70 | SH1C | 14.89478 | 105.4687 | 165 |
| 71 | NS2 | 15.35555 | 105.4549 | 121 |
| 72 | SK6 | 14.54888 | 102.9404 | 265 |
| 73 | SSK2a | 14.57201 | 104.5299 | 194 |
| 74 | HO2 | 14.5497 | 105.0028 | 198 |
| 75 | DH13 | 16.6379 | 106.7703 | 235 |
| 76 | NS1 | 15.2958 | 105.4738 | 130 |
| 77 | DH12 | 16.59594 | 106.6304 | 205 |
| 78 | no code | 16.63887 | 106.7703 | 197 |
| 79 | H11-1 | 16.23427 | 107.2721 | 589 |
| 80 | H11-2 | 16.24335 | 107.2833 | 616 |
| 81 | H11-4 | 16.29027 | 107.3586 | 78 |
| 82 | no code | 16.05458 | 107.4874 | 50 |
| 83 | (KT1) | 15.14218 | 107.7527 | 902 |
| 84 | KT5 | 14.74969 | 107.6826 | 667 |
| 85 | (KT6) | 14.85824 | 107.6969 | 647 |
| 86 | TK11 | 15.38614 | 108.6918 | 16 |
| 87 | TK13 | 15.51508 | 108.3995 | 37 |
| 88 | P1 | 15.10492 | 105.8475 | 141 |
| 89 | no code | 15.34814 | 106.1306 | 868 |
| 90 | T4 | 15.40653 | 106.3859 | 920 |
| 91 | T5 | 15.43438 | 106.4241 | 780 |
| 92 | S1 | 15.38218 | 106.6875 | 188 |
| 93 | S2 | 15.34211 | 106.6989 | 146 |
| 94 | S7 | 15.2315 | 106.745 | 126 |
| 95 | A1 | 14.93514 | 106.8422 | 133 |
| 96 | P2 | 15.10331 | 105.85 | 133 |
| 97 | SK2 Lalu | 14.05194 | 102.575 | 125 |
| 98 | NP2 | 17.47019 | 104.7256 | 156 |
| 99 | NP3 | 17.47074 | 104.7237 | 170 |
| 100 | NP4 | 17.46442 | 104.7253 | 157 |
| 101 | NP9 | 17.47114 | 104.6985 | 142 |
| 102 | M2 | 16.85719 | 104.7433 | 142 |
| 103 | M11 | 16.63609 | 104.7066 | 157 |
| 104 | NW7 | 15.95792 | 105.3536 | 139 |
| 105 | CM3 | 15.06164 | 105.6924 | 145 |
| 106 | P4 | 15.0865 | 105.8932 | 172 |
| 107 | A5 | 14.65181 | 106.851 | 113 |
| 108 | point 134 | 15.11028 | 106.6059 | 674 |
| 109 | Y1 | 15.98194 | 104.0447 | 140 |
| 110 | Point 324 | 14.72975 | 105.344 | 165 |
| 111 | Point 326 | 14.70582 | 105.3552 | 177 |
| 112 | no code | 11.62944 | 109.1633 | 69 |
| 113 | ND2 | 15.31417 | 105.7661 | 128 |
| 114 | ND3 | 15.33 | 105.7917 | 120 |
| 115 | NP1 | 15.60139 | 105.7869 | 146 |
| 116 | NP3 | 15.63083 | 105.7839 | 178 |
| 117 | BXN2 | 15.97611 | 105.4903 | 135 |
| 118 | BXN5 | 16.01861 | 105.6003 | 176 |
| 119 | L6 | 16.2825 | 106.8822 | 600 |
| 120 | no code | 16.25944 | 106.8867 | 606 |
| 121 | L4 | 16.27 | 106.7297 | 381 |
| 122 | EMT4 | 15.96222 | 106.3383 | 182 |
| 123 | EMT1 | 15.95056 | 106.3689 | 204 |
| 124 | NEB1 | 15.3225 | 106.0553 | 925 |
| 125 | NEB2 | 15.32194 | 106.0539 | 922 |
| 126 | NEB5 | 15.32861 | 106.0456 | 930 |
| 127 | TL2 | 15.48472 | 106.2781 | 565 |
| 128 | TT2 | 15.35352 | 106.4926 | 827 |
| 129 | HK1 | 15.10861 | 106.6047 | 676 |
| 130 | SX1 | 14.74694 | 106.5522 | 90 |
| 131 | SX2 | 14.75278 | 106.535 | 88 |
| 132 | P2 | 15.1 | 105.8428 | 128 |
| 133 | MC7 | 14.92639 | 105.8917 | 89 |
| 134 | SOK8 | 14.43694 | 105.7025 | 94 |
| 135 | SOK2 | 14.68861 | 105.7067 | 101 |
| 136 | KST4 | 13.53222 | 105.9144 | 93 |
| 137 | TC11 | 13.67083 | 107.2525 | 212 |
| 138 | TC7 | 13.61194 | 106.8475 | 141 |
| 139 | TC3 | 13.58194 | 106.7931 | 114 |
| 140 | SS2(Sre Sbov) | 12.87889 | 106.1961 | 67 |
| 141 | SS4 | 12.86167 | 106.1939 | 71 |
| 142 | Loc 23 | 13.36722 | 106.0847 | 101 |
| 143 | Krahad | 15.63075 | 102.0021 | 208 |
| 144 | Wang Pong | 16.36209 | 100.8338 | 127 |
| 145 | Mai Chumphon | 15.96445 | 104.0361 | 148 |
| 146 | Bang Nong Makhue | 16.12956 | 102.2196 | 208 |
| 147 | Tad Hua Khon | 15.22745 | 106.74483 | 140 |

^1^ Numbers are those depicted within Figure 3 of the main manuscript

^2^ Northings and eastings refer to the general location where sections were examined. Northings and eastings provided within in the main text are specific sections.

^3^ Altitudes are for guidance only, being derived from a hand-held gps unit or from Google Earth^©^.


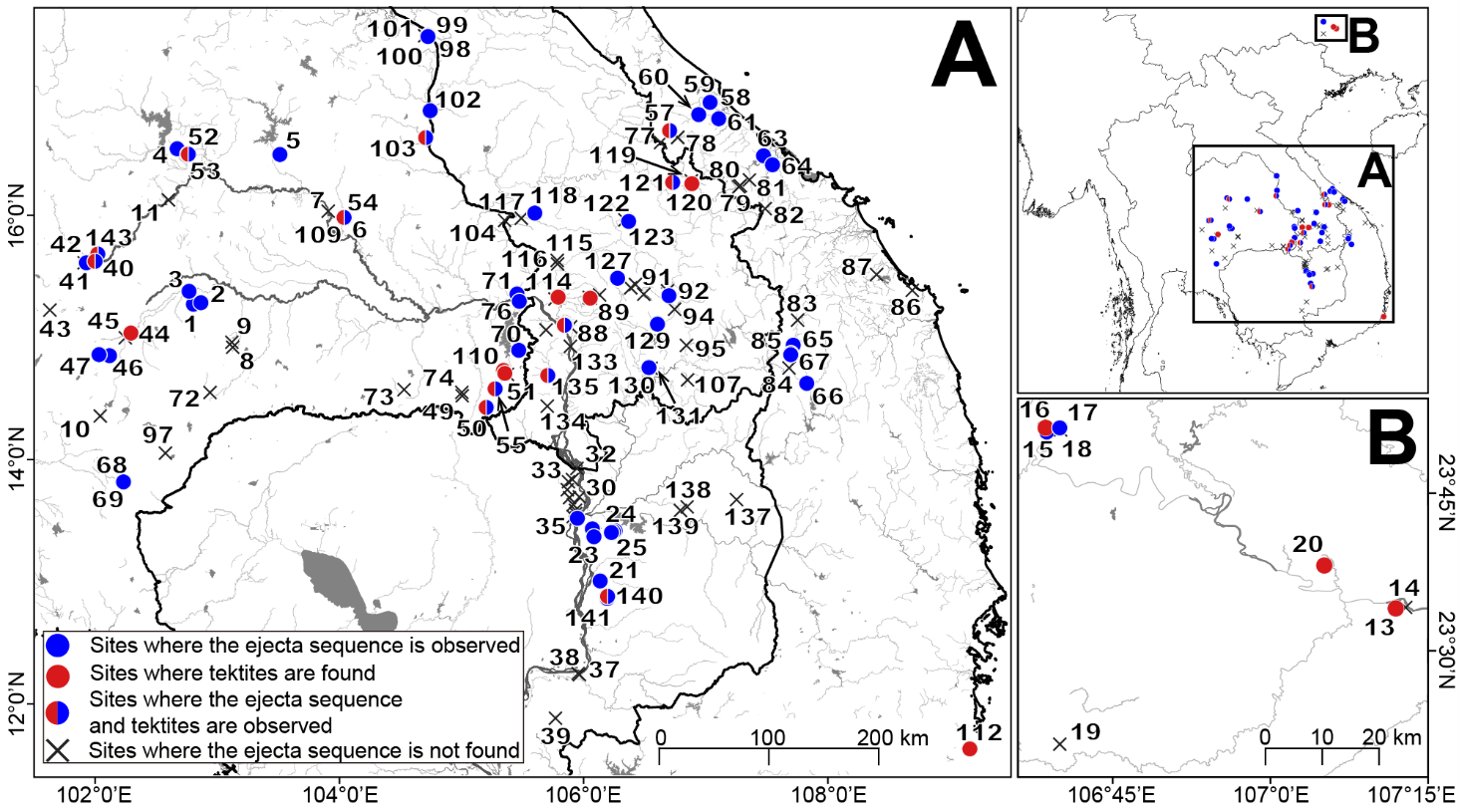


*Figure S1: Locations where sedimentary sections were examined during this project. See Table S2*

**X-ray scans**

Inspecting the X-ray of the basal cover-sand there is no evidence of lamination at all, rather the sediment appears massive although mottled due to diagenesis and heavily bioturbated (Fig. S2).


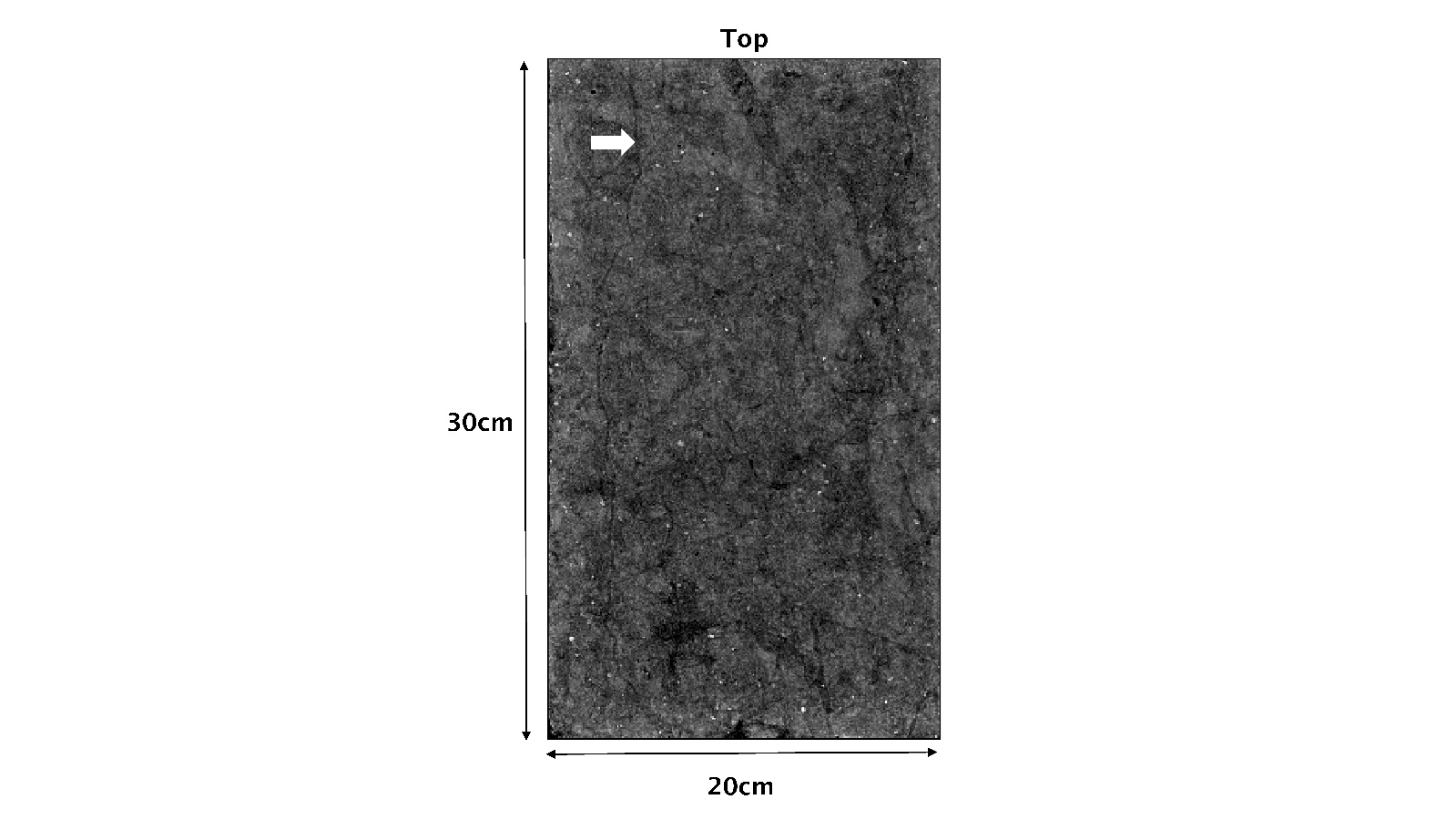


*Figure S1: X-radiograph of a rectangular intact sediment block representing the basal 30 cm height of the red sandy portion of Unit 3 near Krahad, Nong Sa-nga, Chaiyaphum, Thailand. The basal granule layer (just below this image) was not sampled. Note the absence of horizontal lamination, rather the deposit is mottled and heavily bioturbated by rootlet traces, termite and other insect burrows. The white arrow points to a burrow that extends from close to the top of the block to the base. The fine sand appears as grey or black whereas small white ‘pinpricks’ are fractured quartzite granules, which are more concentrated near the base but are increasingly dispersed throughout the remainder of the deposit.*

**Optically-stimulated dating of the stratigraphy**

See Cresswell *et al*., 2018a, b, c; 2019; Creswell *et al*., 2022 for analytical details.

Table S3: Summary of dose rates, luminescence properties, equivalent doses and ages for the three cover sand units at two of the study sites in Thailand (analysis by Geological Survey of Israel).

| Location Code | Unit 3  division | Depth  (m) | Dose rate  (μGy/a) | Aliquots  used | OD  (%) | De  (Gy) | Age  (ka) |
| --- | --- | --- | --- | --- | --- | --- | --- |
| **Krahad** | | | | | | | |
| Krahad-3 | Upper cover sand | 1.1 | 859±36 | 19/19 | 18 | 7.0±1.3 | 8.1±1.6 |
| Krahad-2 | Lower cover sand | 1.5 | 854±37 | 17/19 | 21 | 7.7±0.9 | 9.0±1.1 |
| Krahad-1 | Granule layer | 1.8 | 670±28 | 17/19 | 22 | 5.7±1.0 | 8.5±1.5 |
| **Kok Yai** | | | | | | | |
| Kokyai-3 | Upper cover sand | 0.8 | 876±37 | 18/19 | 20 | 6.5±1.0 | 7.5±1.2 |
| Kokyai-2 | Lower cover sand | 1.4 | 846±37 | 19/19 | 22 | 10.3±2.5 | 12.2±3.0 |
| Kokyai-1 | Granule layer | 1.7 | 645±27 | 19/19 | 21 | 12.4±2.7 | 19.2±4.3 |

Of the 14 sandy-samples taken for OSL dating in this study, six (Table S3) were used to define the relative or absolute ages of the three upper cover-sand units. At both Krahad and Kok Yai, the three units young upwards but the basal granule layer is not as old as expected. Seven further sandy samples, together with two from Table S3, represent samples taken from the basal granule layer across the region. The Tad Hua Khon sample (Table S4) is the exception being a sample of a 0.7-1.0 m thick pebble-sized breccia that lies between the sandstone basement and a basalt flow. All these results show considerable variability in luminescence sensitivity, dose rates and equivalent doses and dose distributions.

Four of the sites yield quartz with high luminescence sensitivity, with little dispersion in the equivalent dose determined on multiple aliquots. Equivalent doses determined by single-aliquot regenerative-dose (SAR) OSL on quartz from these sites range from 4.3 ± 0.1 Gy (Sa Kaeo), 6 ± 1 Gy (Krahad) to 35 ± 2 Gy (Khon Kaen), with the two studies by different laboratories of the Kok Yai site in good agreement (12 ± 4 and 10.7 ± 0.5 Gy). For those sites

Table S4: Summary of dose rates, luminescence properties, equivalent doses and ages for granule layer (Unit 3) and Unit 1 from eight sites in Thailand, Laos and Vietnam, with an additional sample from below a lava flow (analyzed by SUERC, University of Glasgow).

| Location | Dose rate  (mGy a^-1^) | OSL intensity (c Gy^-1^) | Equivalent dose  (Gy) | Age  (ka) | Comment/Analyst |
| --- | --- | --- | --- | --- | --- |
| Khon Kaen, Thailand | 1.0 ± 0.1 |  | 35 ± 2 | 35 ± 2 | Sanderson et.al. (2001) |
| Kok Yai, Thailand | 0.65 ± 0.03 |  | 12.4 ± 2.7 | 19.2 ± 4.3 | Analysis by N. Porat |
| Krahad, Thailand | 0.67 ± 0.03 |  | 5.7 ± 1.0 | 8.5 ± 1.5 | Analysis by N. Porat |
| Kok Yai, Thailand | 1.0 ± 0.1 | 102700 ± 5900 | 10.7 ± 0.5 | 10.7 ± 0.5 | E_D_ from SAR-OSL  Cresswell *et.al.* 2019a |
| Huai Om, Thailand,  Top of Unit 1 | 1.6 ± 0.1 | 1000 ± 100 | 150 ± 20 | 95 ± 15 | E_D_ from TL-ramp & TT-OSL, inflated by 20%  Cresswell *et.al.* 2019a |
| Huai Om, Thailand,  Base of Unit 1 | 2.4 ± 0.1 | 3680 ± 750 | 280 ± 15 | 120 ± 10 | E_D_ from TL-ramp & ID, inflated by 20%  Cresswell *et.al.* 2019b |
| Sa Kaeo, Thailand | 0.5 ± 0.1 | 89200 ± 4100 | 4.3 ± 0.1 | 8.6 ± 0.2 | E_D_ from SAR-OSL  Cresswell *et.al.* 2019a |
| Hue, Vietnam | 2.0 ± 0.2 | 810 ± 90 | 28 ± 2 (OSL)  200-250 (extended) | 14.4 ± 2.1  100-125 | Cresswell *et.al.* 2018a,b |
| Pakse, Laos | 0.8 ± 0.1 | 2330 ± 380 | 120 ± 20 | 150 ± 25 | E_D_ from TL-ramp & TT-OSL, inflated by 20%  Cresswell *et.al.* 2019b |
| Tad Huakhon, Laos.  Below lava flow | 1.5 ± 0.1 | 302400 ± 28300 | 120 ± 25 | 80 ± 20 | E_D_ from TL-ramp & TT-OSL, inflated by 20%  Cresswell *et.al.* 2019b |

where environmental luminescence profiling was conducted (Sa Kaeo, Khon Kaen, Kok Yai and Huai Om) the TL signals produce apparent doses which are similar to the OSL measurements, suggesting significant light exposure prior to deposition resetting both optical and thermal signals. The other two sites yield quartz with luminescence intensities two orders of magnitude lower, in both cases with SAR-OSL resulting in aliquots that saturate (the equivalent doses exceeding the limits of the procedure, greater than 50 Gy). The site at Hue, Vietnam, shows evidence of mixing between younger material (28 ± 2 Gy) and much older material (200-250 Gy). Whereas, at Huai Om there is little evidence of mixing of young and old material with all the aliquots saturating under SAR-OSL, and dose extension methods (TL-ramp and TT-OSL) resulting in relatively narrow dose distributions (150 ± 20 Gy). The profiling results from Huai Om shows a transition between low and high sensitivity quartz, with the top sample an order of magnitude more sensitive. Whereas profiling measurements from Huai Om show a large residual TL apparent dose, suggesting limited light exposure prior to deposition, the profile from Hue shows a low TL apparent dose consistent with the non-saturating OSL equivalent dose.

The profiling data show clear differences between the high and low sensitivity materials, both with at least an order of magnitude difference in OSL intensities (typically ~1000 photon counts for the low sensitivity materials, and ~10,000-100,000 for the high sensitivity) and differences in OSL depletion indices (typically 3.5-4.0 for low sensitivity materials and 4.5-5.5 for the high sensitivity).

Dose rates vary by a factor of four. These materials are generally low in potassium, as observed in high resolution gamma spectrometry measurements conducted at SUERC and the Geological Survey of Israel on the Kok Yai and Krahad samples (see Supplementary Information: TT & OSL analysis) where it was noted that all the Krahad samples and the basal Kok Yai sample had K concentrations below detection limits. The lowest dose rate (0.5 ± 0.1 mGy a^-1^) is at Sa Kaeo in the south of Thailand. Dose rates for the central and northern areas in Thailand are similar in the range 0.7-1.0 mGy a^-1^, and higher in NE Thailand at Huai Om (1.6 ± 0.1 mGy a^-1^). The highest dose rate is at Hue, Vietnam (2.0 ± 0.2 mGy a^-1^). It is noted that these higher dose rate samples, driven by higher K content at ~0.5%, are also the samples which include quartz with equivalent doses more than the OSL-SAR saturation dose (~50Gy). The data indicate that these sandy layers can be divided into at least two groups. One, sands with ~0.5% K including quartz carrying equivalent doses more than 50 Gy (saturating OSL-SAR methods). Two, sands with lower (< 0.2%) K concentrations and quartz carrying equivalent doses less than 50 Gy (not saturating OSL-SAR methods). At Huai Om, both groups are present, with the second group overlaying the first, implying that these are older, rather than the higher equivalent doses being a result of the higher dose rates and potential residual dose. At Hue, there is evidence that there has been mixing between these groups even at the base of the deposits. At the other sites this older group of materials is absent.

For both Huai Om and Hue, the oldest components have similar ages determined from the dose extension analyses (100 ± 15 ka at Huai Om, 100-125 ka at Hue). These are significantly younger than the 700-800 ka age of the meteorite impact resulting in the tektite gravel layer. The analysis of samples from Hue indicated that the traps responsible for the dose extension measurements may not be thermally stable at environmental temperatures in the region, and thus may produce equivalent doses that have been reduced and ages that are less than the physical ages of these sediments. Preliminary measurements of the trap parameters are insufficient to calculate the appropriate kinetics to determine thermal stability.

The ages of the basal layers of the younger group of materials range from 9-18 ka (8.6 ± 1.7 ka for Sa Kaeo, 8.5 ± 1.5 ka for Krahad and 10.7 ± 1.2 ka and 19.2 ± 4.3 ka for the two locations at Kok Yai) to 35 ± 4 ka at Khon Kaen. For the Huai Om profile, the bottom sample of the overlying high sensitivity material has an apparent dose of 48 ± 1 Gy which, assuming the dose rate is similar to the measured dose rate for the gravel layer at Huai Om, would yield an approximate apparent age of 30 ± 3 ka, which is consistent with the age of the basal layer of the high sensitivity material at Khon Kaen. With a factor of four difference in the ages determined for these basal samples in different locations, it appears that either the cover sand deposits were not laid down at the same time across the region, or that in some locations there has been significant mixing of old and young material*.*

The older material sampled at Huai Om and Hue is significantly younger than the expected dates for material associated with the tektites in the laterite layer. It has been previously noted that the stratigraphic ages of many Australite tektites, dated by K-Ar methods to 750-800 ka, recovered from Australia are around 7-24 ka (Lovering et al., 1972, Chalmers et al., 1976, 1979, Glass, 1978). Glass (1978) notes that in other locations, particularly relevant here within Indochina, the stratigraphic ages of tektites correspond to the K-Ar and fission track ages. Koeberl (1992) notes that Muong Nong-type tektites are sometimes deeply eroded, mainly by interaction with water, thus the paradox of tektites with ages of 750-800 ka within much younger sediments could be explained by erosion and re-deposition of the tektites from their original settings within more recent strata, which is at odds with the preservation of ‘wrapped’ and fragile tektites.

If the older material dated here to 100-150 ka does correspond to the stratigraphic settings for the tektites, then this relatively young age could be the result of re-working of the gravel layer moving tektites upwards into younger strata. Or, more likely, the traps used to extend the range of equivalent dose measurements may be unstable over ~10^5^-year timescales. It is noted that these older materials are present in samples from Huai Om and Hue. Broadly similar ‘older’ OSL dates were also recorded by Wang *et al*., (2018) for comparable stratigraphic positions, as noted above, with Wang *et al*., (2018) arguing the age of the basal deposits are older than conventional OSL-dating can indicate.

The Huai Om and Pakse samples have similar characteristics to the other samples from below the young cover sands - low OSL sensitivity and similar equivalent doses from the dose extension methods. Within the precision utilized in this study there is very little difference in the age of these samples, which is pointing towards some other factor (such as trap stability at the environmental temperatures of the region) which is limiting this approach. The Tad Huakhon sample is very different from the cover sands and granule layers, being the only sample from beneath a lava flow, with very much higher OSL sensitivity. The equivalent doses given by the thermal transfer methods are, however, comparable with the other samples giving a similar age.

The luminescence methods applied herein readily distinguish between two distinct classes of material within these sand layers: 1. Sands with lower luminescence sensitivity and OSL depletion indices with ages in excess of ~50 ka (the limit of SAR OSL methods); 2. Sands with high luminescence sensitivity and OSL depletion indices with ages less than 35 ka. At four locations in Thailand only the younger material is present, with ages for basal samples of 9-35 ka. At the Thai site, Huai Om, both are present, with the older material in the breccia (Table 2), and the younger material immediately above the gravel layer giving an age of approximately 30 ka. At the site in Vietnam, Hue, there is evidence that the materials have experienced mixing, with some younger material within the older layer. Dose extension methods have been used to estimate equivalent doses for the older materials. Applied to the younger materials, these give equivalent doses within 20% of the OSL method. For the older materials at Huai Om and Hue these give equivalent doses consistent with an age of 100-150 ka, although the data suggest that the traps associated with these measurements have lifetimes of ~10^5^ -10^6^ years at environmental temperatures of 25 °C, and even lower mean lives at environmental temperatures of 30 or 35 degrees. higher temperatures. In these cases, significantly underestimates of the age of early Quaternary events would be expected at elevated temperatures in the 25-35 °C region. Further investigations into trap stability and age extension may be useful as recent investigations elsewhere have pushed the age determination of TT-OSL back to 715 ka (Faershtein *et al*. 2020).

**Termites & Bioturbation**

The effect of bioturbation on the soil profile primarily is related to termite activity but remains uncertain. Tree fall, root growth and decay must also have been of importance reworking surface soils over the millennia (Waters, 1992, p. 307) although visible root traces in the Yasothon soil are limited to near the surface (Kheoruenromne, 1987). The role of rodents is also relevant, although little understood (Whitford & Eldridge, 2013). When NE Thailand was forested, the density of termite mounds was about 2 - 2.5 per hectare (Pendleton, 1942a; Choosai *et al*., 2009) so bioturbation can be expected to be extensive. However, although some species of termites can burrow to depths of many metres, Fukui (2000) argued that in Thailand bioturbation is insignificant below *c*., 1m. Further, the relevant two species in Thailand do not repeatedly rebuild their mounds. Rather, the mounds are occupied for extended time periods (Pendleton, 1942a). Thus, the degree of reworking of the sandy cover sand by repeated construction of mounds is unclear. Termites frequently select for the clay fractions in their mounds (Lima *et al*., 2018) and the mounds in Thailand are depleted of all coarser fractions in contrast to the near-surface soil (Pendleton, 1942b). However, despite the presence of clay-rich termite mounds, the surface cover sand has not become enriched in clay that could have been brought up by termites from the clay-rich basement. Rather, the cover sand contains *c*., 10 to 15% clay throughout (Pendleton, 1942a; Perry 1992) with clay concentration increasing with depth. Whitford & Eldridge (2013) and Kristensen et al., (2019) argue that thin pisolite and iron-rich gravel layers can occur a few decimetres beneath a thin fine-grained soil due to termites selectively moving fines upwards from a shallow depth (< 1.5m), which may explain the presence of shallow, thin, laterites in the study area. These iron-rich layers can be stone lines developed by termites reworking the soil and concentrating stones (Young, 1976) but in SE Asia stone lines are few and lie at shallow depths well above the reworked gravel layer, Unit 2.

There are nine arguments against the cover sand being bioaccumulated:

1. The dispersed nature of many granule bands, whereby matrix-supported granules are scattered within a defined band which exhibit sharp upper and lower boundaries, suggests a primary deposition mechanism.
2. The marked lateral variation (*e.g*., < 2m) from an occasional thick layer of granules that are grain-contact supported to a dispersed layer is difficult to relate to bioconcentration.
3. The distinct thickening and thinning of the granule layer lying within synclines to anticlines developed at the top of the underlying gravel impact breccia (often over short distances) is difficult to relate to bioconcentration.
4. At some locations (*e.g.,* P1) a dispersed granule layer underlies a coarse, compact, grain-contact breccia, so biogenic reworking is not possible through such a layer.
5. There can be marked lateral variation in the number of distinct granule bands (typically 1 to 3; *e.g*., at P1) for which no evident bioaccumulation mechanism exists.
6. At some locations, distinctive lamination (*e.g*., at P1) of cover-sand interbedded with granule bands has not been disrupted by biogenic reworking.
7. The granule layer commonly occurs at considerable depth, beneath the usual depth of biogenic reworking.
8. There is no evidence of insect or mammal burrows, or root traces at the depths of the granule beds. X-ray and CT scans show no evidence of bioturbation.
9. The environmental luminescence data show an intimate relationship between the granule layer and the pebble bed below.

**References**

Cresswell, A.J., Sanderson, D.C.W., Carling, P.A. (2018a) *Luminescence Profile Measurements on Samples from Vietnam Submitted by P. Carling*. Technical Report. SUERC, East Kilbride, UK. <http://eprints.gla.ac.uk/249340/>

Cresswell, A.J., Sanderson, D.C.W., Carling, P.A. (2018b) *Dose Extension of a Sample at the Base of a Sedimentary Sequence in Vietnam*. Technical Report. SUERC, East Kilbride, UK. <http://eprints.gla.ac.uk/249342/>

Cresswell, A.J., Sanderson, D.C.W., Carling, P.A. (2018) *Luminescence Analyses of Samples from Thailand and Laos*. Technical Report. SUERC, East Kilbride, UK.

Cresswell, A.J., Sanderson, D.C.W., Carling, P.A., Darby, S. (2019) *SE Asia Agricultural Soils Age Analysis*. Technical Report. SUERC, East Kilbride, UK. <http://eprints.gla.ac.uk/249339/>

Cresswell, A.J., Sanderson, D.C.W., Carling, P.A., Darby, S. (2022) Quartz age extension applied to SE Asian cover sands. *Quaternary Geochronology*, 69, 101271. <https://doi>.org/10.1016/j.quageo.2022.101271

Choosai, C., Mathieu, J., Hanboonsong, Y., Jouquet, P. (2009) Termite mounds and dykes are biodiversity refuges in paddy fields in north-eastern Thailand. *Environmental Conservation*, 36, 71–79. https://doi.10.1017/SO376892909005475

Fukui, H. (2000) The Budel’s double-planation theory and its applicability to the Khorat Plateau. pp 1-29. In: International Seminar on Landform Evolution and the Environmental Change of Northeast Thailand, Khon Kaen, 18-19 December 2000.

Kheoruenromne, I. (1987) Red and yellow soils and laterite formation in the northeast plateau, Thailand. *Chemical Geology,* 60, 319-326. https://doi.org/10.1016/0009-2541(87)90138-0

Kristensen, J.A., Boetius, S.H., Abekoe, M., Awadzi, T.W., Breuning-Madsen, H. (2019) The combined effect of termite bioturbation and water erosion on soil nutrient stocks along a tropical forest *catena* in Ghana. *Catena*, 178, 307-312. https://doi.org/10.1016/j.catena.2019.03.032

Lima SS, Pereira MG, Pereira RN, Pontes RM, Rossi CQ. (2018) Termite mounds effects on

soil properties in the Atlantic Forest biome. Revista*Brasileira de Ciência do*Solo, 18, 42: e0160564.

<https://doi.org/10.1590/18069657rbcs20160564>

Pendleton, R.L. (1942a) Some results of termite activity in Thailand soils. *Thai Science Bulletin*, 3, 29-53 plus figures and plates.

Pendleton, R.L. (1942b.) Importance of termites in modifying certain Thailand soils. *Journal of the American Society Agronomy*, 34, 340- 344. https://doi.org/10.2134/agronj1942.00021962003400040005x

Sayngam, C., Punyarungka, T., Pratormbut, V. (2013) Sedimentology and provenance of Yasothon soil series, Noen Sa-nga district, Chaiyaphum Province, Joint Batchelor of Education (Physics) thesis, Nakhon Ratchasima Rajabhat University, 154pp.

Waters, M.R. (1992) Principles of Geoarchaeology: a North American Perspective. University of Arizona Press, Tucson.

Whitford, W.G., Eldridge, D.J. (2013) Effects of ants and termites on soil and geomorphological processes. In: Shroder, J. (Editor in Chief), Butler, D.R., Hupp, C.R. (Eds.), Treatise on Geomorphology. Academic Press, San Diego, CA, vol. 12, Ecogeomorphology, pp. 281–292.

Xiao, W., Hongbing, J., Shijie, W., Huashuo, C., Changshun, S. (2014) The formation of representative lateritic weathering covers in south-central Guangxi (southern China). *Catena*, 118, 55–72. https://doi.org/10.1016/j.catena.2014.01.019

Young, A. (1976) Tropical Soils and Soil Survey, Cambridge University Press, Cambridge, 468pp.

Yuan, B., Hou, Y., Wang, W., Potts, R., Guo, Z., Huang, W. (1999) On the geomorphological evolution of the Bose basin, a lower Paleolithic locality in south China*. Acta Anthropologica Sinica*, 18, 215-224. https://www.anthropol.ac.cn/EN/Y1999/V18/I03/215
